# Plant Polyphenols as Heart’s Best Friends: From Health Properties, to Cellular Effects, to Molecular Mechanisms of Action

**DOI:** 10.3390/ijms26030915

**Published:** 2025-01-22

**Authors:** Sanja Stankovic, Slavica Mutavdzin Krneta, Dragan Djuric, Verica Milosevic, Dragan Milenkovic

**Affiliations:** 1 Center for Medical Biochemistry, University Clinical Center of Serbia, 11000 Belgrade, Serbia; 2 Faculty of Medical Sciences, University of Kragujevac, 34000 Kragujevac, Serbia; 3 Institute of Medical Physiology “Richard Burian”, Faculty of Medicine, University of Belgrade, 11000 Belgrade, Serbia; slavica.mutavdzin@gmail.com (S.M.K.); dragan.djuric@med.bg.ac.rs (D.D.); 4 Department of Anatomy, Faculty of Medicine, University of Niš, 18000 Nis, Serbia; verica.milosevic@gmail.com; 5 Department of Food, Bioprocessing and Nutrition Sciences, Plants for Human Health Institute, North Carolina State University, Kannapolis, NC 28081, USA; 6 Department of Nutrition, University of California Davis, Davis, CA 95616, USA

**Keywords:** polyphenols, heart health, in vivo study, in vitro study, bioavailability

## Abstract

Polyphenols are micronutrients found in fruits, vegetables, tea, coffee, cocoa, medicinal herbs, fish, crustaceans, and algae. They can also be synthesized using recombinant microorganisms. Interest in plant-derived natural compounds has grown due to their potential therapeutic effects with minimal side effects. This is particularly important as the aging population faces increasing rates of chronic diseases such as cancer, diabetes, arthritis, cardiovascular, and neurological disorders. Studies have highlighted polyphenols’ capacity to reduce risk factors linked to the onset of chronic illnesses. This narrative review discusses polyphenol families and their metabolism, and the cardioprotective effects of polyphenols evidenced from in vitro studies, as well as from in vivo studies, on different animal models of cardiac disease. This study also explores the molecular mechanisms underlying these benefits. Current research suggests that polyphenols may protect against ischemia, hypertension, cardiac hypertrophy, heart failure, and myocardial injury through complex mechanisms, including epigenetic and genomic modulation. However, further studies under nutritionally and physiologically relevant conditions, using untargeted multigenomic approaches, are needed to more comprehensively elucidate these mechanisms and firmly prove the cardioprotective effects of polyphenols.

## 1. Introduction

### 1.1. What Are Polyphenols?

Polyphenols are secondary metabolites characterized by one or more hydroxyl groups (–OH) attached to aromatic phenols [1,2,3]. While they are predominantly plant metabolites, polyphenols are also found in fish, crustaceans, and algae, and can be synthesized by recombinant microorganisms [1,2,4,5]. In modern medicine, synthetic drugs remain the cornerstone for treating numerous diseases due to their rapid efficacy. However, these drugs are often associated with various adverse side effects [6]. This limitation has prompted an urgent global effort among scientists to investigate natural compounds derived from plants and microorganisms. These compounds hold promise for disease prevention and treatment, often exhibiting minimal side effects compared to synthetic alternatives [7,8,9,10].

Given their diverse structural and pharmacological properties, along with their widespread presence in dietary sources, polyphenols have become a significant focus of scientific research. Studies employing in vitro conditions and animal models have explored their potential health benefits [3,11,12,13]. To date, over 8000 polyphenols have been identified [14], with active biomolecules detected in a variety of foods, including fruits (apples, almonds, cashews, walnuts, and berries), vegetables, dark chocolate, olive oil, grains, medicinal herbs, mushrooms, and red wine [2]. Polyphenols exhibit a broad spectrum of beneficial properties, such as antioxidant, anti-inflammatory, anti-allergic, anticancer, antihypertensive, and antimicrobial activities. Consequently, they play a critical role in preventing cardiovascular and neurodegenerative diseases, as well as certain types of cancer [2].

The daily consumption of polyphenol-rich foods (e.g., fruits, vegetables, and legumes), moderate intake of red wine (one to two glasses), regular physical activity, stress reduction, spirituality, and social connections have been identified as lifestyle factors contributing to the longevity and health quality observed in “blue zones” such as Okinawa (Japan), Sardinia (Italy), Ikaria (Greece), Nicoya (Costa Rica), and Loma Linda (California, USA) [15].

Polyphenols are broadly categorized into two groups: non-flavonoids and flavonoids [16]. Non-flavonoids are further divided into subgroups such as stilbenes, lignans, phenolic acids, and tannins based on their carbon structures [17]. Flavonoids, on the other hand, include flavanones, flavones, flavonols, flavanols, isoflavones, and anthocyanidins, all of which share a common C6–C3–C6 structural framework [16].

#### 1.1.1. Non-Flavonoids

Stilbenes (C6–C2–C6) are polyphenolic biomolecules synthesized by plants such as grapes, peanuts, berries, wine, nuts, and certain fruits, primarily as a response to stress (Table 1). Among natural stilbenes, resveratrol (3,4′,5-trihydroxystilbene) is the most extensively studied. It is found in the red seaweed Jania rubens [3], grapes, and red wine [2,18]. Resveratrol has been investigated for its potential role in managing various disorders. Interest in this compound initially arose from the “French paradox”, which describes the improved cardiovascular outcomes observed in the French population despite a diet rich in saturated fats [19]. Research has demonstrated that resveratrol, along with related stilbenes such as oxyresveratrol, polydatin, pterostilbene, and viniferin, exhibits significant antiproliferative, antioxidant, and anti-inflammatory effects. These properties position them as promising candidates for therapeutic applications in human medicine [6]. However, further studies are required to address the potential toxicity and optimize their clinical use.

Lignans (C6–C3–C3–C6), a subgroup of polyphenols, are complex polymers formed by the coupling of two phenylpropanoid units [20]. Key lignans include secoisolariciresinol diglycoside and matairesinol, which are predominantly found in the seed coat of flaxseeds [21]. For optimal absorption, flaxseeds should be consumed in ground form [22]. Lignans possess antioxidant properties and act as phytoestrogens, contributing to the alleviation of menopausal symptoms and reducing the risk of heart disease, breast cancer, and osteoporosis [23]. Sesame seeds are another notable source, containing sesamin, a lignan with diverse biological benefits [24]. Despite their therapeutic potential, optimizing methods for lignan extraction and refinement remains necessary for their application in dietary supplements and pharmaceuticals [20]. Lignans exhibit anticancer, antioxidant, anti-estrogenic, and antimutagenic activities, highlighting their importance in disease prevention [25]. They are primarily found in flax and sesame seeds, with lesser amounts present in pumpkin seeds, sunflower seeds, cereals, algae, potatoes, legumes, fruits, and most vegetables, nuts, and whole-grain products [3,21,24] (Table 1).

Phenolic Acids (C6–C1 and C6–C3) are a class of polyphenols characterized by the presence of a carboxylic acid group [26]. They are abundant in red fruits, vegetables, tea, coffee, and cereals (Table 1). Despite their prevalence, phenolic acids have often been overlooked in scientific research, even though they are the dominant polyphenols in many plants, particularly in roots. These compounds play a crucial role in the formation of plant-microbe symbiosis [27]. Phenolic acids exhibit a wide range of health benefits, including antioxidant, anticancer, antidiabetic, antimicrobial, anti-inflammatory, and neuroprotective effects [8,26,28]. Studies suggest their antioxidant activity surpasses that of well-known antioxidants like vitamins C and E [29], making them valuable for promoting a healthier diet and as potential therapeutic agents. Their natural metabolism by microbes enhances their applicability as safer alternatives to harmful synthetic chemicals [2].

Tannins are water-soluble polyphenolic compounds known for their resistance to biodegradation [30]. They are present in a variety of plant-based foods, many of which have a relatively low nutritional value. Rich sources of tannins include red fruits, black and green tea, grape seeds, pomegranate skins and seeds, chestnut skins and leaves, and dark chocolate enriched with cocoa (Table 1) [31,32,33]. Tannins exhibit antimicrobial properties, effectively inhibiting the growth of bacteria, viruses, fungi, and yeasts [22,32]. Tannic acid and propyl gallate, for instance, have been shown to inhibit waterborne and foodborne bacteria, as well as microorganisms responsible for off-flavors in food products. In the food industry, the antimicrobial properties of tannins are utilized to extend the shelf life of processed foods, including fish fillets. Certain types and doses of tannins also have medicinal applications, as they modulate the immune response, accelerate blood clotting, lower blood pressure and serum lipid levels, and demonstrate antimutagenic effects [32].

#### 1.1.2. Flavonoids

Flavonoids (C6–C3–C6) are a subclass of polyphenols that have garnered significant scientific interest in recent decades due to their pivotal roles in numerous biological processes and vital cellular functions [34]. Structurally, flavonoids comprise two aromatic rings connected by a three-carbon bridge and one or more hydroxyl groups. They are classified into six subclasses (Table 1): flavanones, flavones, flavonols, flavanols, isoflavones, and anthocyanidins [7,35].

Flavanones, a distinct subclass of flavonoids, are primarily found in citrus fruits and occur as both aglycones and glycosides [7]. Notable representatives include naringenin, predominant in grapefruit, hesperetin in oranges, and eriodictyol in lemons [7,35] (Table 1). Animal studies involving aged rats treated with lemon extract have demonstrated enhanced liver antioxidant systems, which help mitigate potential oxidative imbalances [36]. Extensive research has highlighted the beneficial effects of citrus flavanones on human health, including their anti-inflammatory and anti-arthritic properties (particularly naringenin) and their ability to improve thyroid dysfunction in aging populations [37,38].

Flavones, another flavonoid subclass, are highly light-sensitive compounds. Key representatives include apigenin and luteolin, typically found in food as glycosides [17]. Rich dietary sources of apigenin include spices such as parsley, celery, and thyme, as well as certain yellow and orange fruits and vegetables, particularly hot peppers (Table 1) [39]. Studies have shown that flavones, particularly apigenin and luteolin, play significant roles in autophagy and exhibit protective effects against Alzheimer’s disease, depression, diabetes, and insomnia [40]. Apigenin has demonstrated potent anticancer properties by suppressing cancer cell proliferation while maintaining low cytotoxicity, making it a promising candidate for cancer prevention [39]. However, further multidisciplinary research and clinical trials are essential in order to ensure its safe integration into clinical practice [41].

Flavonols, characterized by a hydroxyl group at the 3-position, are also known as 3-hydroxyflavones. Prominent representatives include quercetin, myricetin, and kaempferol [42]. Quercetin, one of the most extensively studied flavonols, has been associated with improved insulin secretion, a reduced risk of type 2 diabetes, the mitigation of free radical damage, significant reductions in plasma triglycerides, and a decreased risk of coronary heart disease [43]. Dietary sources of quercetin include onions, apples, peaches, broccoli, red peppers, grapefruit, tomatoes, potatoes, walnuts, and tea (Table 1) [8]. Kaempferol, known for its anticancer and anti-inflammatory effects, has shown particular importance in cardiovascular protection [44]. Foods rich in kaempferol include broccoli, cabbage, kale, beans, leeks, tomatoes, strawberries, and grapes [44]. Despite the promising health benefits of flavonols, further multidisciplinary studies are required to determine optimal dosages and dietary forms tailored to specific conditions to avoid potential adverse effects [43].

Flavanols (flavan-3-ols), represented by catechin, epicatechin, and epigallocatechin, are predominantly found in cocoa, dark chocolate, and berries [45,46]. Cocoa-derived products, particularly dark chocolate, are especially rich in catechins and epicatechins [17]. Flavanols are unique in that they occur exclusively as aglycones in food [45]. Long-term flavanol supplementation has been shown to improve metabolic parameters, including blood pressure, lipid profiles, blood glucose levels, body mass index, and antioxidant activity, collectively reducing the risk of metabolic syndrome [47].

Isoflavones, nonsteroidal phenolic compounds and a subclass of flavonoids, are structurally similar to 17β-estradiol and are commonly referred to as phytoestrogens [48]. Bioactive isoflavones include aglycones such as genistein, daidzein, biochanin A, and glycitein, along with their glucosides daidzin, genistin, and glycitin [17,49]. Soy and soy-based products are the primary dietary sources of phytoestrogens [49]. Other sources include legumes, beans, onions, melons, carrots, green leafy vegetables (e.g., broccoli and cabbage), and fruits such as apples, pineapples, apricots, cherries, and citrus fruits (Table 1) [50]. Soy isoflavones have been extensively studied for their health benefits, particularly in middle-aged and elderly individuals, where they have been shown to reduce risk factors for coronary heart disease [51]. Additionally, phytoestrogens have been linked to decreased incidence of breast, prostate, and colorectal cancers [43,52], as well as lower body mass, and increased bone mineral density in postmenopausal women [53]. Despite their health benefits, phytoestrogens are classified as endocrine disruptors, defined by the World Health Organization as exogenous substances or mixtures that alter endocrine system function and cause adverse effects in organisms, progeny, or populations [54]. Thus, further research is required to establish safe dosages and usage guidelines that account for variations in age, gender, and ethnicity [48].

Anthocyanidins, another flavonoid subclass, are water-soluble pigments responsible for the coloration of flowers and fruits [55]. Representatives include cyanidin, malvidin, delphinidin, and peonidin, which are found in blackcurrants, red raspberries, strawberries, chokeberries, plums, pomegranates, oranges, beans, cabbage, and red onions (Table 1) [56]. Anthocyanins, the glycosylated forms of anthocyanidins, have been shown to positively influence cholesterol and lipoprotein metabolism in patients with dyslipidemia [17]. Anthocyanidins and anthocyanins exhibit neuroprotective effects and play roles in preventing diabetes, cardiovascular diseases, and bacterial infections [57]. Numerous in vivo, in vitro, and clinical studies have highlighted their anti-tumor potential, mediated through mechanisms such as anti-mutagenesis, antioxidant activity, anti-inflammation, cell cycle arrest, and the induction of apoptosis or autophagy in cancer cells [58]. Furthermore, anthocyanins possess anti-inflammatory, anti-obesity, and lipid-modulating properties [59]. Meta-analyses have demonstrated that both anthocyanins and anthocyanidins can significantly reduce the risk of mortality from coronary heart disease and cardiovascular diseases [60]. Flavonoids (C6–C3–C6) belong to the class of polyphenols, and, in recent decades, have been the focus of scientific research, because they play an important role in numerous biological processes and vital functions in the cell itself [34]. They consist of two aromatic rings connected by three carbon atoms and one or more hydroxyl groups and can be divided into six subclasses (Table 1): flavanones, flavones, flavonols, flavanols, isoflavones, and anthocyanidins [7,35].

### 1.2. Metabolism and Bioavailability of Polyphenols

Polyphenols are dietary micronutrients that play a crucial role in preventing age-related diseases, particularly cardiovascular, metabolic, and neurodegenerative disorders [21]. A diet rich in polyphenols, sourced from fresh vegetables, fruits, cereals, tea, coffee, and red wine, promotes overall health and contributes to healthy aging, owing to their antioxidant, anti-inflammatory, and anti-cancer properties [2,7]. However, the health effects of polyphenols are influenced by their consumption levels and bioavailability, which can vary significantly. The most abundant dietary polyphenols may not always exhibit the highest bioavailability [61]. Understanding their impact on human health is complex, as their biological activity is often altered by metabolism, leading to ambiguous and sometimes contradictory findings among researchers [16].

Polyphenol bioavailability is affected by several factors, including sex, age, food matrix interactions, liver-mediated metabolic processes, gut microbiota, and the metabolites formed in vivo [16,62,63]. For instance, the bioavailability of lignans from flax and sesame seeds can be enhanced by grinding the seeds before consumption, as the seed coat is otherwise difficult to digest [24]. Flavanols exhibit higher bioavailability compared to other flavonoids [63]. Conversely, proanthocyanidins and anthocyanins show low bioavailability, limiting their effects primarily to the intestines [62].

Dietary flavonoids are predominantly present as glycosides, which must undergo deglycosylation in the small intestine prior to absorption [64]. Aglycones and certain glucosides are readily absorbed in the small intestine [65], while non-deglycosylated flavonoids are hydrolyzed by β-glucuronidase in the large intestine, followed by reabsorption or fecal elimination [61,66]. Free aglycones are subsequently conjugated by phase II enzymes in the intestine and liver, resulting in sulfated and glucuronidated metabolites detectable in plasma [62]. During absorption, polyphenols undergo methylation, sulfation, and glucuronidation, both in the small intestine and the liver [61].

Recent research highlights the gut as the primary site for metabolizing complex polyphenols into smaller phenolic compounds through microbial activity [67]. For example, (−)-epicatechin, after partial metabolism in the small intestine, undergoes further transformation by gut microbiota in the colon, yielding 5-(3′,4′-dihydroxyphenyl)-γ-valerolactone metabolites (gVLM), which are present in circulation and are believed to mediate the beneficial effects of (−)-epicatechin intake [68]. Similarly, unabsorbed anthocyanins in the small intestine are biotransformed by the gut microbiome. Glycosylated anthocyanins with disaccharide moieties are more resistant to digestion compared to mono-glucosides and aglycones [69]. Microbial enzymes in the large intestine cleave sugar bonds, hydrolyzing intact anthocyanins [69]. The resulting aglycones are unstable and further metabolized into phloroglucinol aldehyde and phenolic acids, derived from the A-ring and B-ring structures, respectively [70]. In the presence of colonic bacteria, phloroglucinol aldehyde is oxidized to form phloroglucinol acid [71].

These findings underscore the significant role of the gut microbiota in metabolizing polyphenols, enhancing their bioavailability, and facilitating their absorption through the colonic epithelium. Such insights emphasize the intricate relationship between dietary polyphenols, the gut microbiota, and human health, warranting further investigation in order to optimize their therapeutic potential.

## 2. Polyphenols and Cardiovascular Health

According to data from the World Health Organization (2023) [72], there is an alarming rise in the prevalence of diabetes and cardiovascular diseases, particularly among the elderly population, with 19.5 million deaths reported annually. The primary contributors to the high disease burden in older individuals are unhealthy lifestyles and habits [73]. Key factors for a healthier lifestyle include dietary modifications, increased physical activity, smoking cessation, and adjustments in daily routines. These measures are fundamental for preventing aging-related diseases such as cardiovascular disorders, type 2 diabetes mellitus, dementia, and certain cancers [74]. Over the past 50 years, the Mediterranean diet—prevalent in Southern Europe and the Levant [75]—has emerged as an optimal dietary model for promoting healthy aging and reducing the disease burden in older age [76]. This dietary pattern emphasizes a higher consumption of fruits, cheese, fish, and red wine, along with a reduced intake of animal fats [77]. Clinical trials have demonstrated that incorporating flavonoid-rich foods, particularly those containing flavanones, into the diet can significantly lower blood triglycerides and cholesterol levels [78]. Furthermore, epidemiological studies consistently highlight the health benefits of polyphenols, underscoring their positive impact on human well-being [79,80].

The remarkable physiological potential of polyphenols in supporting health and preventing disease has fueled growing scientific interest in understanding their mechanisms of action and therapeutic applications. This increasing focus reflects the critical role of polyphenols in promoting longevity and mitigating the effects of aging.

### 2.1. Knowledge from In Vitro Studies

The cardioprotective effects of various polyphenols on cardiac function have been extensively investigated in both in vivo and in vitro models.

A wealth of in vitro studies has been conducted to elucidate the mechanisms of action and cellular effects of polyphenols. These cell-based experiments provide a controlled environment to study the direct interactions between polyphenol compounds and specific cell types, signaling pathways, and molecular targets. By employing a range of cell lines and primary cell cultures, researchers have been able to dissect the impact of polyphenols on processes such as oxidative stress, inflammation, apoptosis, and cellular metabolism. The insights gained from in vitro models are complemented by findings from animal studies, allowing for a more comprehensive understanding of the biological activities of polyphenols at the cellular and molecular levels. Together, in vivo and in vitro approaches are of great importance in uncovering the therapeutic potential of these natural compounds for various conditions and diseases.

**Figure 1:**
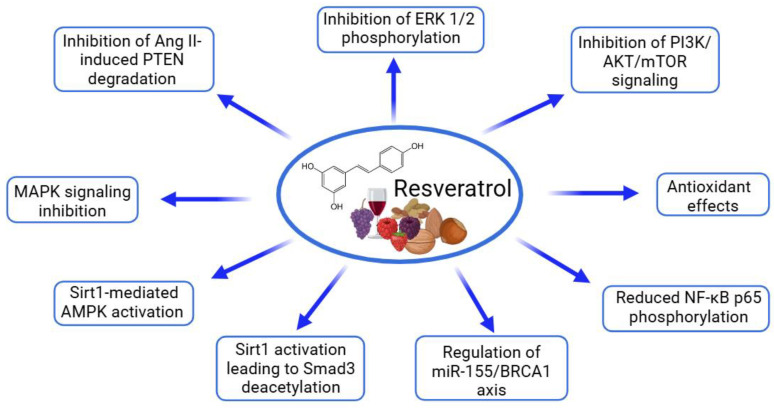
Mechanisms of action of resveratrol in cell models.

Among the tested polyphenols, resveratrol has garnered the most attention, with numerous studies analyzing its impact on cardiac health (Table 2, Figure 1). In vitro experiments frequently utilize H9C2 cardiomyoblast, cardiomyocyte, and cardiac fibroblast cultures to elucidate resveratrol’s cellular mechanisms. Resveratrol has shown significant cardioprotective properties across these experimental models. In H9C2 cardiomyoblasts, resveratrol counteracts the doxorubicin-induced cytotoxicity by enhancing cell viability, reducing lactate dehydrogenase (LDH) release, and mitigating oxidative stress, which includes a reduction in reactive oxygen species (ROS) and lipid peroxidation. Notably, resveratrol inhibits ferroptosis by decreasing intracellular iron (Fe^2+^) levels and restoring glutathione (GSH), while modulating proteins essential to ferroptosis, such as glutathione peroxidase 4 (GPX4), prostaglandin endoperoxide synthase 2 (PTGS2), and acyl-CoA synthetase long-chain family member 4 (ACSL4). Furthermore, resveratrol inhibits mitogen-activated protein kinase (MAPK) signaling pathways, reducing extracellular-signal-regulated kinase (ERK), c-Jun N-terminal kinases (JNK), and p38 activity, which are critical for cell survival under stress [81,82]. In neonatal rat cardiomyocytes, resveratrol reduces angiotensin II (Ang II)-induced hypertrophy by decreasing the expression of hypertrophy markers, including atrial natriuretic peptide (ANP), brain natriuretic peptide (BNP), and skeletal α-actin (ACTA1), and by inhibiting the Ang II/Ang II type 1 receptor (AT1R) signaling pathway. Resveratrol’s anti-inflammatory effects are also notable, as it disrupts nuclear factor-kappa B (NF-κB) signaling by preventing the phosphorylation of NF-κB p65 and inhibitor of κB (IκB), limiting NF-κB nuclear translocation and, thereby, reducing inflammation [83,84,85,86]. Additionally, resveratrol attenuates fibrosis in cardiac fibroblasts stimulated with transforming growth factor β1 (TGF-β1) by activating the silent information regulator of transcription 1 (Sirtuin 1—Sirt1), which leads to the reduced acetylation of Smad3, a transcription factor essential for fibrosis signaling, and the downregulation of fibrosis markers. These findings suggest that resveratrol’s anti-fibrotic properties are mediated through the Sirt1-dependent deacetylation of Smad3, highlighting its therapeutic potential in preventing cardiac fibrosis and remodeling [87,88]. These findings across multiple cell types and pathways underscore resveratrol’s versatile cardioprotective role, mainly through reducing oxidative stress, inflammation, and hypertrophic and fibrotic responses, suggesting its therapeutic potential in cardiac health.

**Figure 2:**
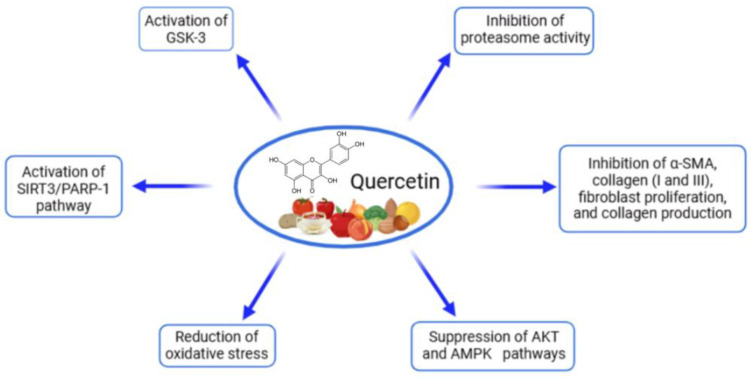
Mechanisms of action of quercetin in cell models.

Quercetin, a flavonoid polyphenol, has been studied for its protective effects on cardiac cells, particularly in models of hypertrophy and fibrosis (Table 3, Figure 2). Studies using in vitro cultures, including H9C2 cardiomyoblasts and neonatal rat cardiac fibroblasts, indicate that quercetin can mitigate the cellular changes associated with cardiac stressors, such as Ang II, a hypertrophic agonist known to induce hypertrophic and fibrotic responses [89,90,91]. In cardiomyocyte cultures, quercetin was shown to reduce the expression of key hypertrophic markers like ANP and β-myosin heavy chain (β-MHC) in a dose-dependent manner. This anti-hypertrophic effect was also associated with improved mitochondrial function, as evidenced by the preservation of mitochondrial membrane potential and enhanced ATP production, largely due to the activation of the silent information regulator of transcription 3 (SIRT3). This pathway appears to protect mitochondria from oxidative damage by regulating the SIRT3/PARP-1 axis, highlighting quercetin’s ability to counteract Ang II-induced mitochondrial dysfunction and oxidative stress [89,90]. Quercetin has also shown efficacy in fibroblast cultures, where it inhibited the proliferation and migration of cardiac fibroblasts and reduced collagen synthesis, crucial steps in the fibrotic process. Additionally, quercetin downregulated the expression of fibrosis markers, including α-smooth muscle actin (α-SMA) and types I and III collagen, which are upregulated in response to Ang II. This suggests that quercetin can hinder fibroblast activation and collagen deposition, thus potentially preventing myocardial fibrosis [91]. These findings collectively indicate that quercetin can modulate multiple signaling pathways in cardiac cells, including those involved in hypertrophy and fibrosis, suggesting its promise as a therapeutic agent to protect against adverse cardiac remodeling.

**Figure 3:**
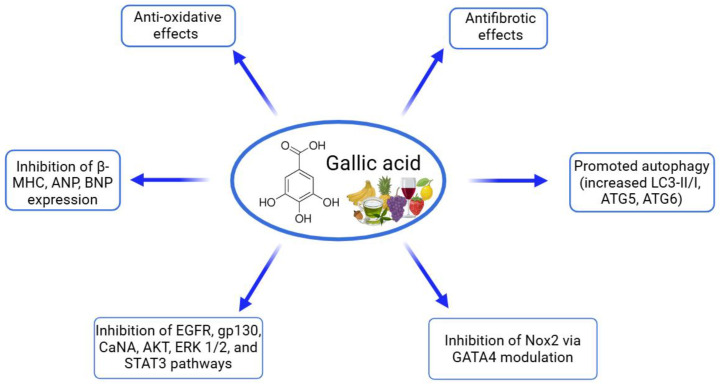
Mechanisms of action of gallic acid in cell models.

Gallic acid, a phenolic compound known for its antioxidant and anti-inflammatory properties, has shown promising protective effects against cardiac hypertrophy and fibrosis in various cell culture models (Table 4, Figure 3). Research indicates that gallic acid can counteract hypertrophic responses and fibrotic remodeling in cardiomyocytes and cardiac fibroblasts, particularly when these cells are stimulated with hypertrophic or fibrotic agonists [92,93,94,95]. In studies using neonatal rat cardiomyocytes, gallic acid was shown to significantly reduce hypertrophy induced by Ang II or isoproterenol (ISP) by decreasing the cell size and downregulating the expression of hypertrophic markers, including ANP, BNP, and β-MHC. The compound’s action on these cells also involved autophagy activation, as indicated by the increased levels of autophagic markers LC3-II/I, ATG5, and ATG6, along with reduced signaling through pro-hypertrophic pathways like epidermal growth factor receptor (EGFR), glycoprotein 130 (gp130), and calcineurin (CaNA). This regulation ultimately inhibited hypertrophic signaling cascades, including protein kinase B (AKT), extracellular signal-regulated kinase (ERK) 1/2, and STAT3 pathways, which are critical for cellular growth and stress responses [92,93]. Additionally, studies in H9C2 cardiomyoblasts revealed that gallic acid can attenuate oxidative stress by reducing NADPH oxidase 2 (Nox2) mRNA levels and inhibiting the activation of the Nox2 promoter via GATA4, a transcription factor involved in cardiac remodeling. By lowering ROS production and enhancing nitric oxide levels, gallic acid supports cellular antioxidant defenses, which are critical in combating oxidative damage associated with cardiac hypertrophy and fibrosis [94]. Gallic acid also demonstrated anti-fibrotic effects in TGF-β1-stimulated neonatal rat cardiac fibroblasts, where it effectively reduced levels of collagen type I, fibronectin, connective tissue growth factor (CTGF), and α-SMA. This reduction in fibrotic markers points to gallic acid’s ability to inhibit fibroblast activation and extracellular matrix deposition, processes central to cardiac fibrosis. Immunocytochemistry further confirmed a decreased collagen I expression after gallic acid treatment, underscoring its role in limiting fibroblast-driven fibrotic remodeling [95]. Overall, these findings illustrate that gallic acid can modulate multiple cellular mechanisms, including hypertrophic, fibrotic, and oxidative pathways, thereby offering protective benefits against the progression of cardiac remodeling in various in vitro cardiac cell models.

Genistein, a phytoestrogen with antioxidant properties, has demonstrated cardioprotective effects in various in vitro studies (Table 5). Research on neonatal rat cardiomyocytes and H9C2 cells highlights its potential to mitigate cardiomyocyte hypertrophy, a condition often associated with cardiac stressors like phenylephrine and isoproterenol. In vitro studies using neonatal rat cardiomyocytes demonstrated that genistein at a concentration of 20 μM effectively inhibited phenylephrine-induced hypertrophy. It specifically prevented the hyper-phosphorylation of JNK1/2, a critical signaling pathway involved in cardiomyocyte growth, while not affecting the overall protein levels of MAPK and AKT pathways. This suggests that genistein may modulate hypertrophic responses through targeted signaling inhibition [96]. The treatment of H9C2 cells with 10 μM isoproterenol for 12 h led to notable cell enlargement and a corresponding rise in the hypertrophic markers, while the microRNA (miR)-451 levels decreased significantly. Genistein alone did not induce cardiac hypertrophy or alter levels of hypertrophic markers but significantly increased miR-451 expression. The role of miR-451 was further elucidated through experiments demonstrating that the overexpression of miR-451 inhibited H9C2 cell hypertrophy by targeting the tissue inhibitor of metalloproteinases 2 (TIMP2), a protein involved in cardiac remodeling. The inhibition of miR-451, on the other hand, led to an increased cell size and elevated levels of ANP, BNP, miR-199, and miR-499. These findings underscore miR-451’s role in hypertrophy suppression and highlight genistein’s therapeutic potential through the modulation of microRNA pathways in myocardial hypertrophy induced by pathological stimuli such as isoproterenol, suggesting that genistein may represent a promising strategy for preventing cardiac cells stress [97].

Other polyphenolic compounds have shown considerable potential for cardioprotection, with various in vitro studies revealing their ability to modulate critical pathways involved in cardiac hypertrophy, oxidative stress, and fibrosis (Table 6). Specific compounds such as epigallocatechin-3-gallate, tanshinone IIA sulfonic sodium, luteolin, kaempferol, delphinidin, and caffeic acid phenethyl ester demonstrated diverse mechanisms of action that underscore their promise in treating cardiac dysfunctions [98,99,100,101,102].

In a recent study, polyphenols such as epigallocatechin-3-gallate and tanshinone IIA sulfonic sodium have shown promising cardioprotective effects when incorporated into glycocalyx-like coatings for cardiac occluders. In vitro experiments using H9C2 cardiomyoblasts and endothelial cells demonstrated that the polyphenol-infused coatings significantly enhanced cell adhesion and reduced apoptosis. The release of epigallocatechin-3-gallate and tanshinone IIA sulfonic sodium from the coating exhibited potent free-radical-scavenging abilities, providing antioxidant protection to the cells. Additionally, RNA sequencing revealed that the coatings regulated genes involved in apoptosis, inflammation, and cellular metabolism, particularly through pathways such as NF-κB, tumor necrosis factor (TNF), cyclic adenosine monophosphate (cAMP), and MAPK signaling, highlighting their role in modulating critical cellular processes [98].

In vitro experiments with luteolin at 16 μM on isolated cardiomyocytes from heart failure rats showed significant improvements in contractile function. Luteolin enhanced peak shortening, contraction, and relaxation rates, likely mediated through the upregulation of sarcoplasmic reticulum Ca2+-ATPase (SERCA2a) activity via the PI3K/Akt pathway. Additionally, luteolin promoted SERCA2a stability through sumoylation, offering protection against cellular degradation and improved calcium handling, which is essential for maintaining cardiac contractility under heart failure conditions [99].

Kaempferol (25 µM) has also displayed cardioprotective effects in vitro, specifically in H9C2 cardiomyocytes, where it countered the increase in cell surface area induced by phenylephrine treatment. This effect was attributed to the inhibition of the ASK1/MAPK signaling pathway, reduction in ROS accumulation, and downregulation of apoptosis-related proteins such as Bcl2 and BAX, thereby reinforcing kaempferol’s role as a protective agent against oxidative stress, inflammation, and apoptosis in cardiac cells [100].

Similarly, delphinidin, at concentrations of up to 50 μM, protected cardiomyocytes and cardiac fibroblasts from Ang II-induced hypertrophy, oxidative stress, and fibrotic changes. Cardiomyocytes treated with delphinidin displayed a reduced cell size and decreased expression of hypertrophic genes, such as Anp, Bnp, and β-MHC, along with the inhibition of ROS production and NADPH oxidase (NOX) activity. The obtained data suggest that delphinidin inhibits NOX activity through the activation of AMP-activated protein kinase (AMPK). In vitro, the Ang II-induced upregulation of NOX subunits (specifically p47phox and Rac1) was inhibited by delphinidin, which concurrently increased AMPK phosphorylation. Blocking AMPK with compound C reversed these effects, leading to restored ROS production, NOX activity, and cardiomyocyte hypertrophy, highlighting the essential role of AMPK in delphinidin-mediated cardioprotection. Delphinidin’s inhibition of MAPK kinase phosphorylation in the Ang II-induced hypertrophy models further supports the role of MAPK signaling in its antihypertrophic effects. In cardiac fibroblasts, delphinidin countered the Ang II-induced cell proliferation, migration, and expression of fibrotic markers, such as collagen I, collagen III, and CTGF [101].

The caffeic acid phenethyl ester was shown to reduce hypertrophy in H9C2 cardiomyoblasts exposed to phenylephrine. The caffeic acid phenethyl ester, at 20 μM, prevented increases in cell surface area and inhibited the mitogen-activated protein kinase /extracellular signal-regulated kinase (MEK/ERK) pathway, evidenced by the reduced phosphorylation of MEK1/2 and ERK1/2, critical mediators of the hypertrophic response. This downregulation indicates the caffeic acid phenethyl ester’s potential to mitigate hypertrophy and oxidative stress, highlighting its potential as a therapeutic compound for managing cardiac hypertrophy and related dysfunctions [102]. According to the previous studies results, polyphenols demonstrate robust cardioprotective effects by modulating key molecular pathways involved in hypertrophy, fibrosis, oxidative stress, and inflammation. Their actions in in vitro models indicate the significant potential to counteract cellular damage induced by cardiac stressors, enhancing cell survival, reducing hypertrophic and fibrotic markers, and mitigating oxidative injury. Through specific mechanisms, including the regulation of ferroptosis, activation of Sirtuins, modulation of microRNAs, and inhibition of critical signaling pathways (e.g., MAPK, NF-κB, and PI3K/Akt), these polyphenols offer promising therapeutic avenues to prevent adverse cardiac remodeling. These findings collectively underscore the potential of polyphenols as preventive or adjunctive treatments in cardiac health, warranting further research to confirm their efficacy in clinical settings.

### 2.2. Knowledge from In Vivo Studies

Polyphenols have garnered significant research interest for their potential therapeutic applications in various disease states. To evaluate their efficacy, numerous in vivo studies have been conducted using animal models. These preclinical investigations play a crucial role in elucidating the mechanisms of action, pharmacokinetics, and dose-dependent effects of different polyphenol compounds.

**Figure 4:**
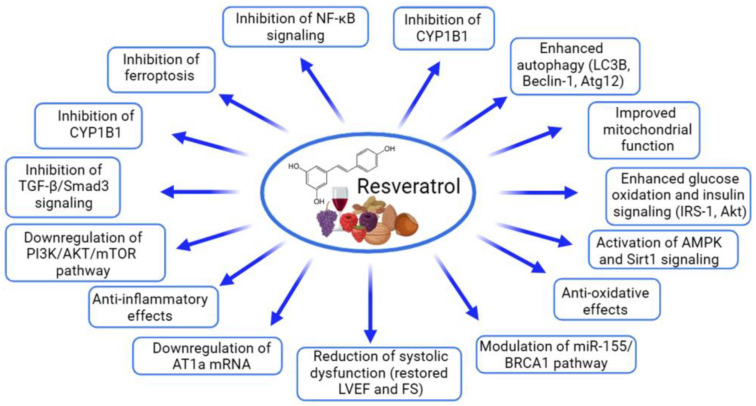
Mechanisms of action of resveratrol in animal models.

**Figure 5:**
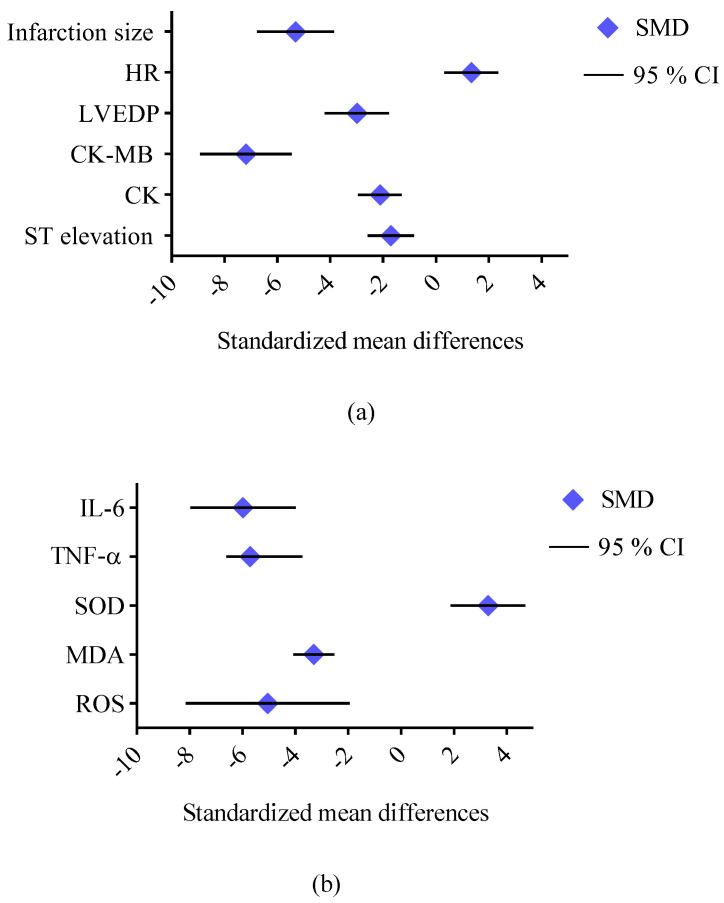
The standardized mean differences (SMD) and 95% confidence intervals (95% CI) for (**a**) the myocardial injury and cardiac function parameters and (**b**) the oxidative stress and inflammation parameters in the meta-analysis of analyses of resveratrol’s cardioprotective effects by Zhang et al., 2024 [108]. CK—creatine kinase; CK-MB—creatine kinase–myocardial band; LVEDP—left ventricular end-diastolic pressure; HR—heart rate; ROS—reactive oxygen species; MDA—malondialdehyde; SOD—superoxide dismutase; TNF-α—tumor necrosis factor-alpha; IL-6—interleukin-6.

Resveratrol has garnered significant attention in preclinical studies for its cardioprotective properties across a range of heart conditions (Table 7, Figure 4). Recent studies have highlighted the potential of resveratrol (in doses ranging from 20 to 450 mg/kg per day) as a cardioprotective agent, demonstrating its ability to improve cardiac function, reduce cardiac remodeling, and mitigate oxidative stress, inflammation, and fibrosis in various preclinical models of heart disease [81,82,83,84,85,86,87,88,103,104,105,106,107]. In mice subjected to doxorubicin-induced cardiac dysfunction, resveratrol (20 mg/kg/day for two weeks) significantly attenuated heart damage, preserving the left ventricular function as evidenced by the improved ejection fraction (EF) and fractional shortening (FS). It also mitigated myocardial tissue damage, reducing cytoplasmic vacuolization and myocardial fiber disarray, while decreasing iron accumulation and GSH levels. These effects were attributed to resveratrol’s inhibition of ferroptosis [81]. Similarly, in a mouse model of Ang II-induced cardiac hypertrophy, resveratrol (45.51 mg/kg/day for 28 days, orally) improved cardiac function and reduced hypertrophy, as shown by the decreases in heart weight/body weight and the heart-weight-to-tibia-length ratios, and mitigated interstitial fibrosis and cardiomyocyte enlargement. Resveratrol effectively inhibited the Ang II/AT1R-induced activation of NF-κB signaling in the heart [86]. In models of myocardial ischemia–reperfusion injury, resveratrol (30 mg/kg/day for 7 days) reduced the infarct size, improved cardiac function (EF and FS), and attenuated the histopathological damage by preserving the myocardial fiber structure and reducing cardiomyocyte apoptosis. The compound decreased oxidative stress in the myocardium and reduced the expression of the pro-inflammatory cytokines tumor necrosis factor-alpha (TNF-α) and interleukin (IL)-1β in cardiac tissue. Furthermore, resveratrol’s activation of the AMPK and Sirt1 signaling pathways contributed to the increased expression of autophagy-related proteins (LC3B, Beclin-1, and Atg12) and decreased levels of p62 [85]. In mouse and rat models of heart failure induced by transverse aortic constriction (TAC), resveratrol (25–450 mg/kg/day) improved the left ventricular function, reduced hypertrophy, and decreased fibrosis, collagen deposition, the hypertrophic markers ANP, BNP, and β-MHC, and oxidative stress. The treatment improved cardiac performance by reducing left ventricular dilatation and enhancing diastolic function. Resveratrol prevented the inactivation of AMPK and improved glucose oxidation rates, which was linked to restored mitochondrial function and improved myocardial energetics. Moreover, resveratrol inhibited the degradation of PTEN and downregulated the activation of the AKT/mTOR signaling pathway while promoting AMPK activation [83,84,103]. Additionally, resveratrol improved insulin sensitivity in skeletal muscle and enhanced vascular function, promoting greater physical activity and potentially improving exercise tolerance. Resveratrol significantly improved the survival of mice with HF. The treatment enhanced whole-body glucose utilization and improved skeletal muscle insulin signaling. Furthermore, resveratrol treatment restored the skeletal muscle oxidative capacity, and these beneficial effects were associated with significant alterations in the gut microbiome [103,104]. In male C57BL/6 mice with heart failure with a preserved ejection fraction, resveratrol (10 mg/kg/day for four weeks) effectively reduced left ventricular hypertrophy and diastolic dysfunction, as well as alleviated inflammation and oxidative stress. Resveratrol also exhibited strong anti-inflammatory properties, reducing the plasma and cardiac tissue levels of pro-inflammatory cytokines including IL-1β, IL-6, and TNF-α, as well as limiting the infiltration of neutrophils and macrophages in the heart. Moreover, resveratrol significantly attenuated cardiac fibrosis by inhibiting the TGF-β/Smad3 signaling pathway [88]. In a rat model of heart failure induced by the left anterior descending (LAD) coronary artery ligation, resveratrol (2.5 mg/kg/day for 16 weeks) improved cardiac function, as shown by the increased left ventricular EF (LVEF) and reduced BNP levels, and enhanced survival. Mechanistic studies in Sirt1 knockout mice revealed that the cardioprotective effects of resveratrol were Sirt1-dependent [82]. In a rat model of myocardial infarction (MI)-induced heart failure, resveratrol (5.82 mg/kg/day for two weeks, orally, starting three weeks post-MI) improved cardiac function, reduced left ventricular and atrial remodeling, and restored fatty acid oxidation. Notably, resveratrol also inhibited the expression of CYP1B1, an enzyme responsible for producing cardiotoxic hydroxyeicosatetraenoic acid (HETE) metabolites [87]. Resveratrol has also demonstrated synergistic effects when combined with other therapies, such as atorvastatin, in rat models of ISO-induced myocardial hypertrophy. Resveratrol (20 mg/kg day) reduced cardiac injury markers and improved antioxidant levels, while decreasing lipid peroxidation [105]. Additionally, in a rat model of chronic intermittent hypoxia (CIH), resveratrol (30 mg/kg daily for five weeks) reversed CIH-induced myocardial hypertrophy, improved cardiac function, protected against cardiomyocyte apoptosis, and mitigated oxidative stress by downregulating the PI3K/AKT/mTOR pathway [106]. The study of cardiac hypertrophy induced by abdominal aortic banding in rats revealed that resveratrol (1 mg/kg/day, for 14 days, ip) significantly inhibited Ang II levels and downregulated the AT1Ra mRNA expression in cardiac tissue, disrupting the renin–angiotensin system (RAS) and preventing Ang II-driven hypertrophy [107]. A meta-analysis of 43 studies investigating the effects of resveratrol in rat models of myocardial ischemia–reperfusion injury provides robust evidence of its cardioprotective properties. Resveratrol significantly reduced markers of myocardial injury, including ST segment elevation (standardized mean difference (SMD) = −1.71, 95% confidence interval (95% CI) [−2.59, −0.84]), creatine kinase (CK, SMD = −2.11, 95% CI [−2.94, −1.29]), and the creatine kinase–myocardial band (CK-MB, SMD = −7.19, 95% CI [−8.92, −5.46]). Hemodynamic improvements included reduced left ventricular end-diastolic pressure (LVEDP, SMD = −2.99, 95% CI [−4.21, −1.77]) and an increased heart rate (HR, SMD = 1.34, 95% CI [0.31, 2.36]). Resveratrol also mitigated oxidative damage by lowering ROS (SMD = −5.05, 95% CI [−8.15, −1.95]) and MDA (SMD = −3.31, 95% CI [−4.08, −2.53]) while enhancing SOD (SMD = 3.29, 95% CI [1.87, 4.70]). Furthermore, it reduced inflammatory markers, including TNF-α (SMD = −5.71, 95% CI [−6.61, −3.74]) and IL-6 (SMD = −5.98, 95% CI [−7.97, −3.99]). Notably, resveratrol decreased the myocardial infarction size (SMD = −5.31, 95% CI [−6.77, −3.86]), highlighting its potential to protect cardiac tissue (Figure 5). These findings emphasize the efficacy of resveratrol in alleviating myocardial injury, improving cardiac function, and reducing inflammation and oxidative stress [108]. Overall, the preclinical studies have consistently shown the cardioprotective properties of resveratrol. These findings highlight the potential of resveratrol as a therapeutic agent for various cardiovascular diseases.

**Figure 6:**
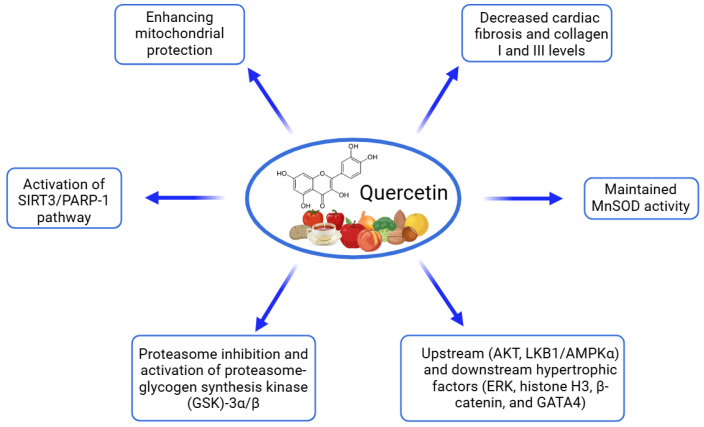
Mechanisms of action of quercetin in animal models.

Quercetin, a flavonoid polyphenol known for its potent antioxidant and anti-inflammatory properties, has been extensively studied in preclinical models for its cardioprotective effects, particularly against cardiac hypertrophy and remodeling (Table 8, Figure 6). Across various studies, quercetin has been used in doses ranging from 5 to 30 mg/kg/day, with treatment durations typically spanning from 8 days to 8 weeks. In models of cardiac hypertrophy and remodeling, quercetin demonstrated significant protective effects through multiple mechanisms. In spontaneously hypertensive rats, quercetin (20 mg/kg/day for 8 weeks) reduced cardiac hypertrophy and fibrosis by activating the SIRT3/PARP-1 signaling pathway, improving mitochondrial function, and enhancing antioxidant enzyme activities [89]. Similarly, in rats with abdominal-aortic-constriction-induced cardiac hypertrophy, quercetin (5, 10, and 20 mg/kg/day for 8 weeks) attenuated left ventricular hypertrophy, improved diastolic function, and reduced cardiac fibrosis by inhibiting elevated proteasome activities [90]. In models of isoproterenol-induced cardiac hypertrophy in mice, quercetin (30 mg/kg/day for 8 days) effectively reversed hypertrophic changes by restoring antioxidant enzyme activity, reducing hydrogen peroxide production, and preventing mitochondrial damage [109]. Furthermore, in Ang II-induced cardiac remodeling in mice, quercetin dihydrate (25 mM/kg every two days for two weeks) reduced heart structural remodeling and improved cardiac function by lowering fibrosis markers, including collagen I and III levels [91]. Additionally, one meta-analysis quantitatively demonstrated the quercetin impact on cardiovascular improvement that was linked to an enhanced cardiac structure in pressure overload (SMD = −1.50; 95% CI: [−2.66, −0.33]; *p* < 0.05; I^2^ = 74.05%) and better systolic and diastolic function in ischemia–reperfusion injury models (SMD = −1.81; 95% CI: [−3.05, −0.56]; *p* < 0.01; I^2^ = 84.93%) [110]. These studies collectively demonstrate that quercetin’s cardioprotective effects are mediated through its antioxidant properties, mitochondrial protection, and modulation of key signaling pathways involved in cardiac hypertrophy and fibrosis, highlighting its potential as a therapeutic agent for cardiovascular diseases.

**Figure 7:**
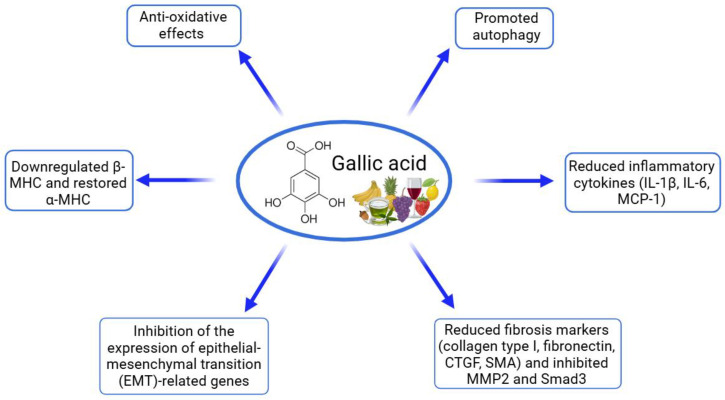
Mechanisms of action of gallic acid in animal models.

Gallic acid, a naturally occurring polyphenol, has demonstrated significant cardioprotective effects across various experimental models of cardiac disease (Table 9, Figure 7). In preclinical studies, gallic acid has been tested at doses ranging from 5 to 100 mg/kg/day, with treatment durations varying from 2 to 16 weeks. Studies demonstrate that gallic acid can significantly improve cardiac function by reducing ventricular diameters, enhancing the ejection fraction, and restoring fractional shortening. Additionally, gallic acid effectively prevents cardiac hypertrophy and fibrosis, as evidenced by the decreased heart weight, reduced collagen deposition, and suppression of pro-fibrotic markers. These protective effects are mediated through multiple pathways, including the attenuation of oxidative stress, inhibition of inflammatory cytokines, and regulation of key hypertrophic and fibrotic signaling pathways, such as MAPK and TGF-β/Smad. Overall, gallic acid shows potential as a therapeutic agent for managing heart conditions linked to chronic stress and pathological remodeling [92,93,94,95,111]. In models of pressure-overload-induced cardiac dysfunction using TAC in mice, gallic acid showed dose-dependent effects. At 5 and 20 mg/kg (oral gavage for eight weeks), gallic acid improved cardiac function by enhancing EF and FS, while reducing cardiomyocyte hypertrophy, fibrosis, and inflammation. The compound decreased oxidative stress markers (NOX2, NOX4, and p22phox) and inflammatory cytokines (IL-1β, IL-6, and monocyte chemoattractant protein-1—MCP-1) [92]. At a higher dose of 100 mg/kg/day for two weeks, gallic acid effectively improved cardiac function by reducing ventricular diameters and restoring FS, while decreasing heart failure markers (ANP and BNP) and regulating hypertrophic gene markers (β-MHC, and α-myosin heavy chain—α-MHC). The treatment also reduced perivascular fibrosis through the downregulation of fibrosis-related markers and the modulation of the TGF-β/Smad signaling pathway [95,111]. In models of ISP-induced cardiac hypertrophy in mice, gallic acid (100 mg/kg/day) demonstrated preventive effects when administered prior to ISP exposure. The treatment reduced cardiac hypertrophy markers, improved fractional shortening, and decreased fibrosis through the inhibition of the MAPK signaling pathway and Smad3 activation [93]. In spontaneously hypertensive rats, gallic acid (320 mg/kg/day for 16 weeks) effectively reduced systolic blood pressure and left ventricular hypertrophy by suppressing cardiac-specific transcription factors GATA4 and GATA6 [94]. These studies collectively demonstrate that gallic acid exerts its cardioprotective effects through multiple mechanisms, including the modulation of hypertrophic signaling pathways, a reduction in oxidative stress and inflammation, and the regulation of fibrotic responses. These findings highlight gallic acid’s potential as a therapeutic agent for various cardiovascular conditions.

Genistein, a naturally occurring polyphenol, has been studied in various animal models of cardiac conditions at doses ranging from 40 to 100 mg/kg/day (Table 10). In research focused on cardiac hypertrophy and remodeling, genistein demonstrated significant cardioprotective effects through multiple mechanisms [96,97]. In a pressure overload model using aortic banding in mice (40 mg/kg/day), genistein reduced the cardiomyocyte size and improved cardiac indices, while decreasing hypertrophic markers (ANP, BNP, and β-MHC) and maintaining healthy cardiac markers (α-MHC and SERCA2α). The compound also showed anti-fibrotic effects by reducing the CTGF and collagen types I and III expression. Echocardiographic measurements confirmed improved left ventricular function and structure [96]. In a different model using isoproterenol-induced cardiac hypertrophy in female ICR mice, genistein (100 mg/kg oral administration) demonstrated antioxidant properties by reducing serum malondialdehyde (MDA) levels and increasing the superoxide dismutase (SOD)/MDA ratio. This study also revealed a potential molecular mechanism through elevated miR-451 expression in cardiac tissue [97]. Across these studies, genistein consistently showed anti-hypertrophic, anti-fibrotic, and antioxidant properties, suggesting its potential therapeutic value in treating cardiac remodeling and dysfunction.

**Figure 8:**
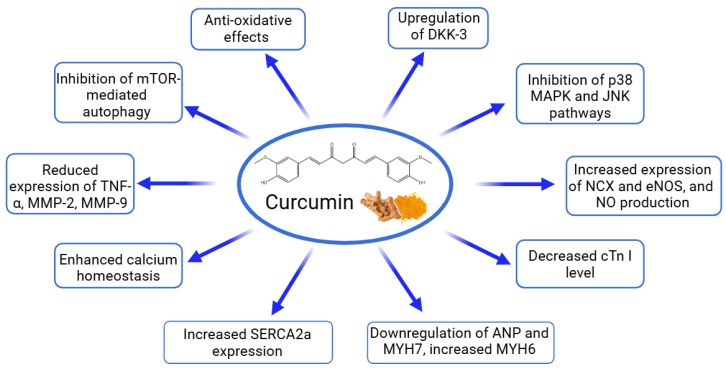
Mechanisms of action of curcumin in animal models.

**Figure 9:**
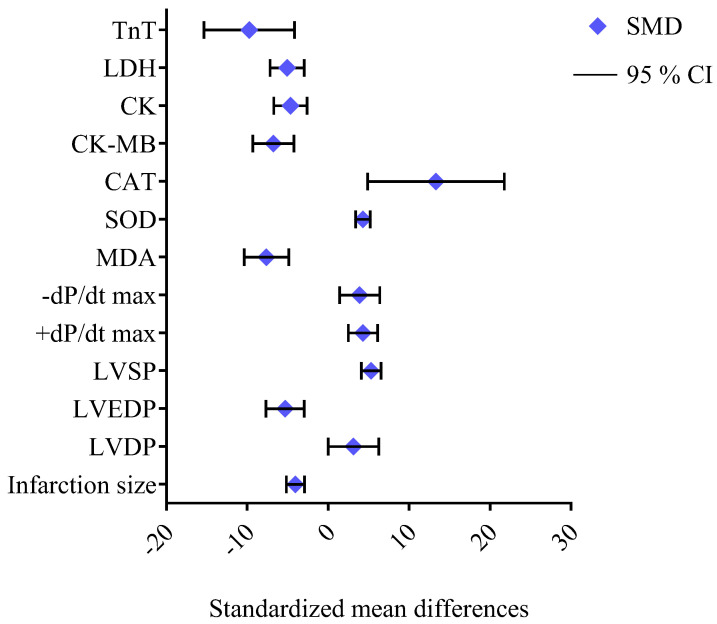
The standardized mean differences (SMD) and 95% confidence intervals (95% CI) according to data from meta-analysis of epigallocatechin gallate’s cardioprotective effects in preclinical models by Wei et al. 2023 [122]. LVDP—left ventricular developed pressure; LVEDP—left ventricular end-diastolic pressure; LVSP—left ventricular systolic pressure; +dP/dt max—maximum rate of rise in left ventricular pressure; −dP/dt max—maximum rate of decline in left ventricular pressure; MDA—malondialdehyde; SOD—superoxide dismutase; CAT—catalase; CK—creatine kinase; CK-MB—creatine kinase–myocardial band; LDH—lactate dehydrogenase; TnT—troponin T.

Curcumin, a polyphenolic compound from turmeric (Curcuma longa), has been extensively studied at doses ranging from 50 to 200 mg/kg/day across various cardiac disease models. The compound’s cardioprotective effects operate through multiple molecular mechanisms and signaling pathways (Table 11, Figure 8). In chronic heart failure induced by volume and pressure overload in rabbits, curcumin (100 mg/kg/day for 18 weeks) improved cardiac function by enhancing LVEF and FS. Mechanistically, it operated through the upregulation of Dickkopf-related protein 3 (DKK-3), which inhibited stress-activated pathways including p38 MAPK and JNK. Additionally, curcumin reduced the expression of key inflammatory and remodeling markers (TNF-α, and matrix metlloproteinases (MMP)-2 and -9) while enhancing SERCA2a expression, crucial for maintaining cardiac contractility [112]. In isoproterenol-induced cardiac hypertrophy in rats, curcumin (200 mg/kg/day for 4 weeks) showed anti-hypertrophic effects by decreasing the heart weight/body weight ratios and modulating key markers (reducing ANP and β-MHC, while increasing α-MHC). It also demonstrated a significant impact on autophagy regulation through the mTOR pathway, reversing ISO-induced increases in autophagic markers LC3 and Beclin-1 while upregulating p-mTOR [113]. In TAC-induced cardiac hypertrophy in Wistar rats, curcumin (50 mg/kg/day for 9 weeks) improved cardiac function while restoring endothelial function through the upregulation of Na+/Ca2+ exchanger (NCX) and endothelial nitric oxide synthase (eNOS), thereby improving calcium homeostasis and nitric oxide signaling [114]. In septic rats, curcumin (200 mg/kg/day for 3 days, ip) demonstrated antioxidant effects by increasing SOD levels and reducing MDA levels, while also lowering cardiac troponin I (cTn I) levels, indicating reduced cardiac damage [115,116]. These studies reveal curcumin’s comprehensive cardioprotective effects through multiple pathways: anti-inflammatory signaling, antioxidant mechanisms, calcium handling, nitric oxide regulation, autophagy modulation, and anti-fibrotic processes. Furthermore, two meta-analyses revealed the cardioprotective effects of curcumin in preclinical animal models of myocardial ischemia/reperfusion injury (Table 12, Figure 9). Curcumin significantly reduced the myocardial infarction size and improved cardiac function parameters, including LVEF, left ventricular FS, left ventricular developed pressure, left ventricular end-diastolic diameter, and left ventricular end-systolic diameter. Hemodynamic improvements also included increased +dP/dt max and −dP/dt max. Oxidative stress markers showed significant reductions in MDA and increases in SOD, catalase (CAT), and GSH. Curcumin significantly reduced inflammatory markers, including IL-1β, IL-6, and TNF-α. It also suppressed NF-κB protein expression. Myocardial injury enzymes (CK, CK-MB, and LDH) were significantly reduced. Lastly, curcumin demonstrated anti-apoptotic effects, reducing the apoptotic index and decreased the area of fibrosis [117,118]. Both meta-analyses emphasize the broad cardioprotective potential of curcumin; however, the high heterogeneity observed in several parameters (I^2^ > 50%) underscores the need for further investigations to ensure the robustness and translational relevance of these results

Another widely used polyphenol is kaempherol. Kaempferol has been studied at doses ranging from 10 to 100 mg/kg across different cardiac disease models, demonstrating significant cardioprotective effects through multiple molecular pathways (Table 13) [100,119]. In a pressure overload model using aortic banding in C57BL/6 mice, kaempferol (100 mg/kg for six weeks, orally) demonstrated anti-hypertrophic and anti-fibrotic effects by reducing cardiac mass indices and the cardiomyocyte size, while decreasing the expression of hypertrophic markers (ANP, BNP, α-MHC, and β-MHC). The compound reduced cardiac fibrosis by downregulating key markers (fibronectin, CTGF, and collagen types I and III) through the modulation of the TGF-β/Smad and MAPK signaling pathways. Additionally, kaempferol enhanced the myocardial antioxidant capacity by elevating the GSH and SOD levels [100]. In diabetic rats with ISP-induced heart failure, kaempferol (10 and 20 mg/kg for 42 days, under oral administration) improved cardiac function through several mechanisms. At the molecular level, it reduced cardiac injury markers (LDH, cTn I, and creatine kinase-MB—CK-MB) while enhancing antioxidant defense through increased SOD, catalase (CAT), and GSH-related enzyme activities and decreased MDA levels. The compound’s anti-inflammatory effects were demonstrated through a reduction in key inflammatory markers (NF-κB, TNF-α, IL-1β, and IL-6). Mechanistically, kaempferol operated through the activation of the PI3K/Akt/GSK-3β pathway, promoting cell survival and reducing apoptosis by decreasing caspase-3 activity and increasing Bcl-2 expression. Its antioxidant effects were mediated through the activation of Nrf2 and the inhibition of NF-κB signaling [119]. Kaempferol demonstrates considerable potential as a cardioprotective agent by targeting key markers of cardiac injury and fibrosis and modulating pathways related to cell survival and oxidative stress. These findings suggest that kaempferol may offer therapeutic benefits for cardiovascular health, particularly in conditions like heart failure and hypertrophy.

Apigenin, a natural flavonoid found in fruits and vegetables, has been studied at various doses and administration routes (20 μg/h infusion to 100 mg/kg oral) in hypertension-related cardiac conditions (Table 14) [120,121]. In hypertensive rats, apigenin administration directly to the paraventricular nucleus (PVN) (20 μg/h for four weeks) demonstrated significant cardiovascular benefits by reducing the mean arterial pressure and heart rate. At the molecular level, it decreased cardiac hypertrophy markers (ANP and BNP) and fibrotic markers (TGF-β1 and CTGF), while reducing the myocardial cell diameter and overall heart weight. The compound showed strong anti-inflammatory effects by decreasing pro-inflammatory cytokines and increasing anti-inflammatory IL-10. Its antioxidant action was evidenced by reduced ROS and increased SOD activity in cardiac tissue, along with an improved neurotransmitter balance in the PVN, leading to a reduced sympathetic tone [120]. In renovascular-hypertension-induced cardiac hypertrophy, apigenin (50 and 100 mg/kg for four weeks) showed dose-dependent benefits, with optimal effects at 100 mg/kg. Mechanistically, it operated through the modulation of metabolic pathways, particularly through the downregulation of HIF-1α and the differential regulation of PPAR pathways. The compound upregulated PPARα and its downstream targets (carnitine palmitoyltransferase 1—CPT-1 and pyruvate dehydrogenase kinase 4—PDK-4) while suppressing proliferator-activated receptor γ (PPARγ)-related pathways, leading to enhanced fatty acid oxidation and reduced lipid accumulation. Additionally, apigenin decreased serum Ang II levels and improved both serum and myocardial free fatty acid profiles [121]. These studies highlight apigenin’s comprehensive cardioprotective effects through multiple mechanisms: blood pressure regulation, anti-hypertrophic signaling, anti-inflammatory pathways, antioxidant effects, and metabolic modulation through PPAR signaling.

Recent studies have highlighted the therapeutic potential of polyphenols in preventing or mitigating cardiovascular diseases, particularly through mechanisms such as reducing oxidative stress, fibrosis, and inflammation, as well as modulating lipid metabolism. The following sections summarize the findings from several studies on the effects of specific polyphenols, including epigallocatechin-3-gallate and tanshinone IIA sulfonic sodium, luteolin, delphinidin, caffeic acid phenethyl ester, hesperidin, pterostilbene, and punicalagin on cardiovascular health [98,99,101,102,122,123,124,125,126].

**Figure 10:**
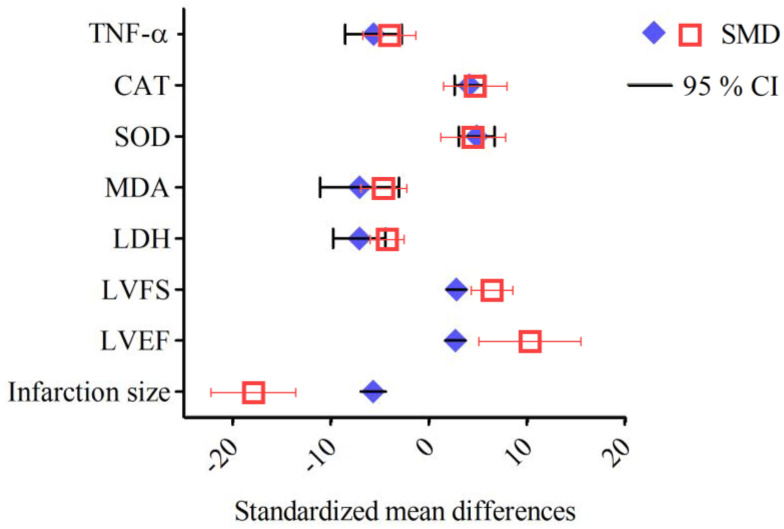
The standardized mean differences (SMD) and 95% confidence intervals (95% CI) according to data from two meta-analyses of curcumin’s cardioprotective effects in preclinical models by Zeng et al. 2023 [117]—blue and Li et al. 2023 [118]—red. LVEF—left ventricular ejection fraction; LVFS—left ventricular fractional shortening; LDH— lactate dehydrogenase; MDA—malondialdehyde; SOD—superoxide dismutase; CAT—catalase; TNF-α—tumor necrosis factor-alpha.

Epigallocatechin-3-gallate and tanshinone IIA sulfonic sodium in glycocalyx-like coatings reduced thrombosis and promoted endothelialization in rabbit and rat models. In rabbits, the coating facilitated endothelial cell migration and reduced inflammation in carotid artery implants, while, in rats, subcutaneous implantation showed reduced fibrosis [98]. One meta-analysis revealed that epigallocatechin gallate demonstrated significant protective effects across multiple cardiac parameters (Figure 10). It substantially reduced the myocardial infarct size and improved various aspects of cardiac function. It decreased LVEDP, increased LVSP, and improved both +dP/dt max and −dP/dt max, while showing a potential effect on decreasing LVDP. Regarding oxidative stress, epigallocatechin gallate demonstrated strong antioxidant properties by decreasing MDA levels and increasing SOD and CAT levels. The compound showed remarkable effects on cardiac enzymes by significantly reducing all measured markers of myocardial injury (CK, CK-MB, LDH, and troponin T—TnT) [122].

Luteolin (10 μg/kg/day) in rats with heart failure induced by abdominal aortic constriction improved left ventricular function and contractility, evidenced by the decreased left ventricular dimensions and increased EF and FS. It reduced cardiac fibrosis, and oxidative stress markers (MDA), while decreasing apoptosis markers Bax and cleaved caspase-3 [99]. According to the meta-analysis luteolin exerts extensive cardioprotective effects in preclinical models of myocardial ischemia–reperfusion injury (Table 15). In the LAD ligation model and the global ischemia model, it significantly reduces the myocardial infarction size, enhances cardiac contractility by increasing left ventricular systolic pressure (LVSP), and improves cardiac relaxation by decreasing left ventricular end-diastolic pressure (LVEDP). It also improves the rate of contraction and relaxation, as reflected by the increased maximum +dP/dt and −dP/dt. Luteolin mitigates oxidative stress by reducing MDA levels, exhibits anti-inflammatory effects by decreasing TNF-α, and reduces the proportion of apoptotic cells, indicating anti-apoptotic activity [123].

Delphinidin (15 mg/kg/day, 8 weeks) in C57BL/6J mice with TAC surgery reduced cardiac hypertrophy (decreased heart weight/body weight and heart weight/tibia length ratios), improved left ventricular function (EF and FS), and decreased ROS and NOX activity. It regulated the hypertrophic gene expression (Anp, Bnp, and β-MHC) and inhibited the MAPK pathway through the reduced phosphorylation of Erk1/2, Jnk1/2, and p38, without affecting mTOR phosphorylation, indicating that MAPK is the primary pathway through which delphinidin mitigates pathological hypertrophy [101].

The caffeic acid phenethyl ester (100 mg/kg/day, 6 weeks) in mice with aortic banding reduced cardiac hypertrophy (heart weight/body weight, and heart weight/tibia length ratios) and improved cardiac function (EF, FS, and end-systolic and end-diastolic diameters). It reduced collagen deposition and downregulated collagen types I and III mRNA by inhibiting the MEK/ERK and TGF-β/Smad pathways, reducing the phosphorylation levels of ERK1/2 and the activation of Smad proteins [102].

Hesperidin (200 mg/kg/day) in rats with ISO-induced myocardial injury preserved left ventricular function, normalized hemodynamic parameters (systolic, diastolic, and mean arterial pressures), reduced oxidative stress markers (MDA), and enhanced antioxidant activity (GSH, SOD, and CAT). It decreased cardiac injury markers (CK-MB and LDH) and caspase-3, increased Bcl-2, and reduced TUNEL-positive apoptotic cells [124].

Pterostilbene (25–100 mg/kg) in rats with monocrotaline-induced pulmonary arterial hypertension improved right ventricular systolic function and cardiac output. It reduced oxidative stress by increasing the GSH-to-glutathione-disulfide (GSSG) ratio, restored antioxidant enzyme activities, and enhanced calcium handling through SERCA upregulation [125].

Punicalagin (250–500 mg/kg, 12 weeks) in LDLR–/– mice on a high-fat diet reduced atherosclerotic plaque formation by 42–57% and aortic root plaque areas by 60–75%. It lowered plasma cholesterol and triglycerides, reduced macrophage infiltration and foam cell formation, decreased inflammatory markers (IL-1β, IL-6, TNF-α, MCP-1, and iNOS), and improved endothelial function through enhanced NO bioavailability and Smad1/5 pathway inhibition. Additionally, it inhibited TNF-α-induced vascular smooth muscle cell migration [126].

These polyphenols demonstrate significant cardioprotective effects through various mechanisms, including a reduction in oxidative stress, inflammation, fibrosis, and hypertrophy (Table 16). These findings support the potential therapeutic application of polyphenols in treating cardiovascular diseases. As natural compounds, these polyphenols offer promising strategies for the prevention and management of heart-related conditions, with further research needed to explore their clinical applicability.

### 2.3. Knowledge from Clinical Trials

Following the high number of in vitro and in vivo studies on the cardioprotective effects of plant polyphenols on different animal models of cardiac disease, global clinical trials have been conducted to clarify its effects on specific body functions, to validate their therapeutic profile, bridging the gap between preclinical findings and their potential clinical applications.

About 750 clinical trials registered from 2000 to 2020 were mainly focused on health effects (520), bioavailability (55), or both (175). Regarding outcomes, primary outcomes were lipid profile and blood pressure (139 registered intervention studies) [127]. The most frequently examined plant polyphenols in clinical studies were as follows: resveratrol, quercetin, catechins, and anthocyanins.

Few systematic reviews and meta-analyses explored the effects of resveratrol on the cardiovascular system. The first systematic review and meta-analysis that analyze evidence from RCTs on the efficacy of supplementation with resveratrol on systolic, diastolic, and mean arterial pressure, and pulse pressure, included 17 RTCs and 681 subjects. It showed that the administration of a high dose of resveratrol (>300 mg/day) increased the systolic and diastolic blood pressure, and a more pronounced lowering of blood pressure in patients with diabetes or obesity [128]. The other meta-analysis of Liu et al. (6 studies, 247 subjects) [129] found that a higher dose of resveratrol consumption (≥150 mg/d) significantly decreases the systolic blood pressure. The meta-analysis (subgroup analysis of 10 human studies with 396 subjects) showed that resveratrol has a significant effect on metabolic parameters (glucose and waist circumference) at a dosage of >500 mg and with long-term interventions of ≥10 weeks [130]. Akbari et al. [131]. in a systematic review and meta-analysis of 28 RCTs showed that the supplementation with resveratrol increases flow-mediated dilatation (FMD) levels among patients with metabolic syndrome and related disorders. The other meta-analysis included 21 arms from 17 studies and showed that resveratrol significantly changes the concentrations of FMD and ICAM-1 [132].

Zheng et al. [133] examined the effect of resveratrol supplementation on cardiac remodelling in 80 hypertensive patients. After 6 months, patients who received 400 mg/day resveratrol in addition to standard therapy had alleviated left atrial remodelling and cardiac fibrosis, and improved left ventricular diastolic function.

Theodotou et al. investigated the efficacy of resveratrol (50 mg/day) in 97 patients with essential hypertension, receiving standard therapy. Patients were followed for 2 years. The revastratol treatment led to a reduction in blood pressure [134]. Only few clinical studies examined the effects of resveratrol in populations with heart failure. Magyar et al. [135] randomized 40 post-infarction Caucasian patients into two groups. In the group that received 10 mg/day of resveratrol for 3 months, improved left ventricle diastolic function and endothelial function, and a lowered LDL-cholesterol level, and protection against unfavourable hemorheological changes were noticed. In patients with stable angina pectoris supplemented with 20 mg resveratrol/day for 2 months, a significant reduction in N-terminal pro–B-type natriuretic peptide levels was observed [136]. Sixty patients with symptomatic systolic HF with a reduced ejection fraction who received 100 mg/day of RES supplementation for 3 months showed improved cardiac function and exercise tolerance, with a significantly reduced total and LDL-cholesterol, interleukin-1 and 6, galectin-3, and N-terminal pro–B-type natriuretic peptide concentrations [137]. All these studies established the beneficial effects of long-term resveratrol supplementation, but Marques et al. [138] found that a single dose may also be effective. They showed that 300 mg of resveratrol improved endothelial function measured by FMD.

Wong et al. [139], in 19 obese and hypertensive subjects, evaluated the acute effects of resveratrol consumption on the human circulatory function and found a significant improvement in endothelial function as measured by FMD of the brachial artery. Fujitaka et al. [140] with supplementation by resveratrol also found improved endothelial function measured by FMD in 34 patients with metabolic syndrome.

The first RCT study [141] that evaluated the beneficial effects of quercetin on hypertension was performed in 2007. This double-blind, placebo-controlled, crossover study included 41 subjects (19 with pre-hypertension and 22 with stage 1) who received 730 mg quercetin or placebo for 28 days. A significant reduction in systolic and diastolic blood pressure was noticed in patients with stage 1 hypertension. Few human clinical trials evaluated the beneficial effects of quercetin on hypertension and the lipid profile [142,143,144,145,146].

Serban et al. performed the first meta-analysis (7 randomized controlled trials (RCTs) comprising 9 treatment arms, and 587 patients) that examined the effects of supplementation with quercetin and showed a significant reduction in systolic and diastolic blood pressure with dosages of ≥500 mg/day [147]. Data from a meta-analysis of 18 RCTs (986 participants), examining the effects of supplementation with flavonols, particularly quercetin on blood lipids, showed a notable decrease in the total- and LDL-cholesterol, and triglycerides, and a significant increase in HDL-cholesterol, whereas fasting plasma glucose and blood pressure were significantly reduced. [148]. Sahebkar et al. found that quercetin supplementation significantly reduced triglyceride concentrations at doses of >50 mg/day [149]. A systematic review and meta-analysis of 8 RCTs revealed the efficacy of quercetin supplementation in reducing systolic blood pressure, but did not ameliorate markers of endothelial function (ICAM-1 and VCAM-1) among patients with metabolic syndrome and related disorders [150]. A year later, Huang et al. [151], in a systematic review and meta-analysis that included 17 RCTs and 896 participants, found that quercetin significantly lowered systolic/diastolic blood pressure. Moreover, the consumption of quercetin for 8 weeks or more significantly improved the high-density lipoprotein cholesterol and triglycerides concentrations. A meta-analysis of 16 RCTs with 1575 patients with metabolic syndrome demonstrated that quercetin supplementation significantly decreased total and LDL-cholesterol levels [152]. In 2022, Papakyriakopoulou et al. [153] published a detailed review of published studies investigating the impact of quercetin on the classical cardiovascular risk factors not included in previously mentioned meta-analyses. Recently, a meta-analysis of 46 randomized controlled trials (RCTs) (2494 participants), published between 2000 and 2021, assessed the effects of polyphenol-rich foods and purified food polyphenol extracts on cardiometabolic risk markers (waist circumference, blood glucose, lipid status, inflammation biomarkers, blood pressure, and flow-mediated dilation). Compared with a placebo, polyphenol-rich food significantly reduced systolic and diastolic blood pressure, until polyphenols extracts showed the most significant reduction in total cholesterol and triglycerides [154]. The number of studies evaluating the effect of quercetin in heart failure and coronary artery disease is limited. Kondratiuk et al. [155] found an improvement in diastolic function (E/e’, LV mass index) in patients with gout and essential hypertension treated with quercetin compared to standard therapy. Chekalina et al. [156] investigated the effect of quercetin on hemodynamic and myocardial ischemia parameters in patients with 85 patients with stable coronary artery disease who received standard treatment and found that it improved both systolic and diastolic left ventricular function. Limited RTCs examined the effects of quercetin on vascular aging parameters [157]. In considering further potential clinical applications, the “quercetin paradox” must be taken into account, such as its potential to interact with medications, leading to altered drug bioavailability [158].

The first clinical trial that evaluated the effect of green tea extract (catechin) supplementation during 3 months on cardiovascular risk factors (blood pressure, inflammation, insulin resistance, and oxidative stress), performed in 56 obese, hypertensive patients, indicated a significant lowering of systolic and diastolic blood pressure values compared to the placebo in the end of the follow-up [159]. Supplementation with 843 mg/day of green tea extract in 1075 postmenopausal women significantly the reduced concentrations of total and LDL-cholesterol [160]. Rostami et al. found that catechin-rich cocoa products led to a significant reduction in blood pressure in patients at high cardiovascular risk [161].

In a meta-analysis of 16 RCTs on systolic blood pressure and 15 RCTs on diastolic blood pressure, epicatechin successfully reduced both systolic and diastolic blood pressure values and the effect was dose-dependent (minimal daily intake of 25 mg epicatechin) [162]. Chatree et al. [163] found that epigallocatechin gallate supplementation with 150 mg/day effectively decreased systolic and diastolic blood pressure.

A recently published study, for the first time, investigated the effects of epigallocatechin gallate on blood pressure and the autonomic nervous system, indicated by the 5 min heart rate variability in obese persons. They found that an 8-week treatment with epigallocatechin gallate decreased systolic and diastolic blood pressure, and mean arterial pressure, and increased the sympathetic activity [164].

Oyama et al. [165] found that a high-dose catechin intake during 2 weeks improved the endothelium-dependent vasorelaxation in 30 smokers. In the study of Alafion et al. [166], in 20 healthy males who received different doses of epicatechin, a significant improvement in vascular function (FMD) 1 h after 1 mg/kg epicatechin intake and 2 h after 0.5 mg/kg compared to the placebo was noticed. Kishimoto et al. [167] investigated the associations between green tea consumption and the prevalence of coronary artery disease in 612 patients who underwent coronary angiography. Higher green tea consumption was significantly inversely associated with the prevalence of coronary artery disease and myocardial infarction in Japanese adults compared to lower green tea intake.

Wang et al. [168] in a meta-analysis of 18 studies determined whether an association exists between tea consumption and the risk of coronary artery disease, and found that one cup of green tea consumed daily was associated with a 10% decrease in the risk of coronary artery disease.

Forty-four patients with periphery artery disease included in the COCOA-PAD study [169] consumed 75 mg epicatechin per day in the form of cocoa or placebo, for 6 months. The average six-minute walking distance in the treatment group increased significantly compared to the placebo.

The data on catechin supplementation effects in patients with heart failure are very scarce. A recently published study examined the effects of milk and dark chocolate on heart failure in 20 patients. NT-proBNP levels decreased significantly in patients who took milk and dark chocolate compared to the placebo, and FMD values increased after dark chocolate consumption [170]. Pereira et al. [171] examined the benefits of cocoa-rich chocolate intake in 30 young, healthy adults, measuring cardiovascular function. They proved the intake of higher cocoa chocolate (high content of epicatechin) improves vascular function by reducing central brachial artery pressures and promoting vascular relaxation. Shafabakhsh et al. [172] in a systematic review and meta-analysis of 16 studies found that catechin supplementation significantly increased FMD, and reduced PWV and AI, but did not affect other markers of endothelial function (ICAM-1 and VCAM-1). Grassi et al. [173] were the first to show that black tea ingestion with a daily dose of 100 mg of tea flavonoids (which equals to <1 cup of tea) dose-dependently improved FMD and decreased peripheral arterial stiffness in healthy volunteers.

Few studies showed that anthocyanins can reduce blood pressure. Jennings et al. [174], in a clinical trial that included 1898 subjects treated with an anthocyanin-rich diet, showed a significant reduction in systolic blood pressure and an improvement in central hemodynamic parameters. McKay et al. [175] showed that 65 subjects with grade I hypertension who consumed hibiscus tea (anthocyanin-rich) for 6 weeks had significantly reduced systolic blood pressure compared to the placebo. In subjects with metabolic syndrome after blueberry consumption, decreased systolic and diastolic blood pressure was recorded [176]. Daily freeze-dried blueberry consumption for 8 weeks reduces blood pressure and arterial stiffness in postmenopausal women with pre- and stage 1 hypertension [177]. Emamat et al. [178] examined the effect of dried purple-black barberry in 84 medicated hypertensive patient for two months and noticed that systolic and mean arterial BP decreased significantly compared to the placebo group. Dohadwala et al. [179] treated 99 patients with coronary artery disease with 480 mL cranberry juice (high anthocyanin intake), and found a beneficial effect on vascular function as measured by FMD. Two meta-analyses confirmed that anthocyanins can positively influence CV risk factors [180,181]. One included 22 trials with 1251 healthy subjects/coronary artery disease patients revealed reduced LDL-cholesterol and glucose levels, and improved systolic blood pressure [180]. The other one included 6 trials with 586 subjects with a high cardiovascular risk and the main finding was a reduction in total and LDL-cholesterol, and triglycerides, and increased HDL-cholesterol [181]. The antiplatelet effects of anthocyanins were evaluated in human clinical trials [182,183].

The first study that showed a significant improvement in the 6 min walk test distance with berry consumption in patients with acute myocardial infarction within 24 h of percutaneous coronary intervention has been reported [184]. According to the literature data, anthocyanin supplementation is associated with a reduction in the risk of acute myocardial infarction. In the study which involved 93,699 women, during 18 years of follow-up, a high anthocyanin intake is connected with a 47% reduction in acute myocardial risk in predominantly young women [185]. Higher intakes of fruit-based flavonoids were associated with a 13% lower risk of nonfatal MI in men [186].

Data on the effect of silibinin/silymarin on classical cardiovascular risk factors are limited. Four systematic reviews and meta-analyses were published between 2016 and 2022 that analyzed RCTs investigating silibinin/silymarin effects on metabolic parameters in patients with type 2 diabetes mellitus and/or metabolic disorders. Voroneanu et al. [187] in a systematic review and meta-analysis of 5 RCTs (270 patients) concluded that routine silymarin administration significantly reduce fasting blood glucose levels and HbA1c, and intensive treatment is connected with a decreased risk for nonfatal myocardial infarction. Hadi et al. [188] performed a systematic review and meta-analysis of 7 RCTs (370 patients with type 2 diabetes mellitus) aiming to evaluate the effect of silymarin supplementation on metabolic status and oxidative stress in subjects with type 2 diabetes mellitus. They found that the supplementation with silymarin can decrease fasting blood sugar, HbA1C, insulin, LDL-cholesterol, and malondialdehyde, and increase HDL-cholesterol levels. A meta-analysis of 10 RCTs (620 patients) with dyslipidemia who received Silymarin extract in a daily dose ranging between 280–2100 mg found that silymarin supplementation in combination with other treatments reduced total and LDL-cholesterol and increased the HDL-cholesterol concentration [189]. A meta-analysis by Xiao et al. [190] which included 15 RCTs and 1 prospective study (1358 patients) of type 2 diabetes mellitus and/or dyslipidemia found that the supplementation of silymarin extract in a dose ranging between 105–1000 mg/day may significantly reduce fasting blood glucose, HbA1c, total and LDL-cholesterol, and triglycerides, and increased HDL-cholesterol.

The overview on RCTs not included in the published meta-analysis studies, investigating the impact of silibinin/silymarin on classical cardiovascular risk factors, was described in a recently published comprehensive review of Kadoglou et al. [191].

Azhdari et al. [192], in a meta-analysis of 7 RCTs, showed the benefits of curcumin supplementation on the significant improvement of fasting blood glucose, triglycerides, HDL-cholesterol, and diastolic blood pressure values. A recently published systematic review and meta-analysis (13 RCTs, with 785 participants intervention durations ranging from 4 to 12 weeks) [193] assessed the effects of curcumin supplementation on metabolic, inflammatory, and oxidative stress indices in patients with metabolic syndrome. It showed significant improvements in waist circumference, fasting blood glucose, diastolic blood pressure, HDL-cholesterol, and TNF-a and CRP levels.

Few studies examined the association between polyphenol intake and cardiovascular mortality. The COcoa Supplement and Multivitamin Outcomes Study (COSMOS) [194] evaluated the effect of cocoa intake on cardiovascular prevention among older adults. Cocoa extract reduced cardiovascular death by 27%. Flavonoid supplementation was associated with a lower risk of cardiovascular death and even relatively small amounts of flavonoid-rich foods may be beneficial for decreasing cardiovascular mortality [195]. Grosso et al. [196] found that a high level of flavonoid supplementation was associated with a reduced risk of all-cause mortality when compared to low-level consumption. The PREDIMED [197] trial evaluated whether polyphenol intake is associated with all-cause mortality in subjects at high cardiovascular risk. Those who reported a high polyphenol intake, especially of stilbenes and lignans, showed a reduced risk of overall mortality compared to those with lower intakes. Moreover, some studies suggest that a regular, low-to-moderate intake of polyphenol-rich red wine has a significant beneficial effect in preventing CVDs and reducing CV mortality [198].

## 3. Molecular Mechanisms of Polyphenols and Heart Health

Early research on the cardiovascular benefits of polyphenols primarily focused on their antioxidant properties, particularly their ability to scavenge free radicals. However, over the past decade, evidence has increasingly emphasized their role in modulating genomic and epigenomic processes, shifting the focus from a purely antioxidant mechanism to broader regulatory effects on the gene and protein expression [199,200]. For instance, quercetin has been shown to upregulate the expression of fibroblast growth factor (FGF) and vascular endothelial growth factor (VEGF) genes in human umbilical vein endothelial cells (HUVECs), contributing to vascular health [201]. More recently, circulating metabolites resulting from the colonic metabolism of dietary polyphenols, such as benzene-1,2-diol-3-sulfate, benzene-1,3-diol-2-sulfate, benzene-1,3-diol-6-sulfate, and phenol-3-sulfate, have been observed to modulate the expression of caveolin-1 and solute carrier transporters in human brain microvascular endothelial cells [202]. Polyphenols exert their effects not only on protein-coding genes but also on non-coding RNAs, such as microRNAs (miRNAs) and long non-coding RNAs (lncRNAs), which play crucial roles in gene regulation. For example, metabolites derived from phase II metabolism and the gut microbiome significantly altered the expression of both miRNAs and lncRNAs in primary human brain microvascular endothelial cells stimulated with TNFα and lipids [203,204]. These findings highlight the complex and multilayered regulatory roles of polyphenols at the molecular level. Additionally, polyphenol-rich foods, such as orange juice, have been shown to influence the expression of over 200 proteins in immune cells of healthy volunteers, indicating their systemic effects on gene and protein expression [205]. Furthermore, epicatechin phase II metabolites were demonstrated to induce modifications in DNA methylation patterns in inflammatory-stimulated HUVECs, likely through interactions with DNA methyltransferases (DNMT1 and DNMT3A), further underscoring their epigenetic influence [206].

Despite the substantial evidence for the molecular mechanisms of polyphenols in vascular and brain cells, relatively little is known about their genomic and epigenomic effects in cardiac tissue, particularly under physiologically relevant conditions that account for polyphenol metabolism. Polyphenols have demonstrated cardioprotective effects through diverse mechanisms in various experimental models. For example, catechin was shown to protect against myocardial ischemia/reperfusion injury by downregulating the expression of non-coding RNA (lncRNA) MIAT in myocardial tissue. In hypoxia/reoxygenation-induced myocardial cells, catechin reduced apoptosis, and the protective effects were attenuated by lncRNA MIAT overexpression [207]. Similarly, epicatechin was reported to mitigate the effects of isoproterenol-induced myocardial infarction in rats by decreasing the heart rate, serum cardiac troponin I levels, heart conjugated dienes, serum high-sensitivity C-reactive protein, and plasma total homocysteine. It decreased the gene expression of pro-inflammatory cytokines, such as nuclear factor-κB, tumor necrosis factor-α, interleukin-1β, and interleukin-6, and upregulated the gene expression of the anti-inflammatory cytokine IL-10 [208].

In a mouse model of accelerated aging (SAMP8 mice), a diet rich in olive oil phenolics enhanced the expression of the nuclear factor erythroid 2-related factor 2 (Nrf2) and its downstream targets, including GST, γ-GCS, NQO1, and PON2, in heart tissue, promoting antioxidative and cytoprotective responses [209]. Hesperidin, another polyphenol, was shown to attenuate sodium-fluoride-induced heart injury by regulating the expression of apoptotic genes such as caspase-3, -6, and -9, Bax, Bcl-2, and cytochrome c, as well as genes involved in the PI3K/Akt/mTOR signaling pathway [210]. Similarly, lemon peel extract containing diosmin, biochanin A, hesperidin, quercetin, and hesperetin upregulated the PI3K, AMPK, and FOXO1 gene expression in the liver and pancreas of diabetic rats, suggesting potential systemic benefits in mitigating diabetes-related complications [211]. Epigallocatechin-3-gallate, a well-studied polyphenol, has been shown to exert significant cardioprotective effects. In diabetic rats, epigallocatechin-3-gallate alleviated myocardial contractile dysfunction, hypertrophy, and injury, primarily by activating autophagy via the AMPK/mTOR pathway and suppressing the TGF-β/MMPs pathway [212]. In models of pressure-overload-induced cardiac hypertrophy, epigallocatechin-3-gallate inhibited hypertrophic responses by modulating PSMB5/Nmnat2/SIRT6-dependent signaling and suppressing NF-κB DNA-binding activity [11]. In a mouse model of heart failure, epigallocatechin-3-gallate reduced the expression of SERCA2a at both mRNA and protein levels by decreasing histone acetylation markers (AcH3 and AcH3K9) near the Atp2a2 promoter, thus regulating calcium handling and myocardial function [213]. In vitro studies further underscore the cellular-level impact of polyphenols. For instance, in high-glucose-induced stress models, epigallocatechin-3-gallate reduced the connexin43 protein expression in cardiomyocytes without affecting mRNA levels, an effect attributed to its inhibition of high-glucose-induced miR-1 expression via the p38-MAPK pathway [214,215].

Emerging studies emphasize the multigenomic effects of polyphenols on the heart. In a rat model of chronic heart failure induced by abdominal aortic constriction, epigallocatechin-3-gallate improved systolic function, reduced fibrosis, and inhibited cardiac hypertrophy. A proteomic analysis of heart tissue identified 162 differentially expressed proteins related to cardiovascular disorders, energy metabolism, oxidative phosphorylation, and lipid metabolism [216]. Similarly, in H2O2-induced oxidative stress in H9c2 cardiomyoblasts, epigallocatechin-3-gallate altered the expression of 330 proteins involved in cellular energetics, mitochondrial electron transfer, and Akt signaling pathways [217].

Despite these promising findings, untargeted omics approaches, including genomics, proteomics, and epigenomics, remain underutilized in cardiac research. Consequently, significant gaps persist in our understanding of the molecular mechanisms underlying the cardioprotective effects of polyphenols. Further research using integrative omics methodologies is crucial in order to elucidate fully the pathways through which polyphenols exert their beneficial effects on the heart.

## 4. Conclusions

**Figure 11:**
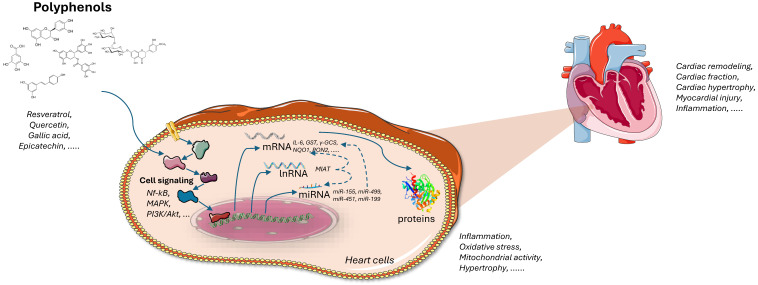
A summary of health effect of polyphenols on the heart from preclinical studies, and heart cells from in vitro studies, together with potential underlying molecular mechanisms of action.

Current evidence demonstrates the cardioprotective potential of polyphenols, including their effects against ischemia, hypertension, cardiac hypertrophy, heart failure, and cellular damage. These benefits have been reported in both preclinical and clinical studies, as well as in vitro (Figure 11). Despite this progress, the molecular mechanisms underlying these protective effects remain incompletely understood and underexplored. Targeted studies highlight the ability of polyphenols to modulate the gene and protein expression in cardiac tissue, influence cell signaling pathways, and induce epigenetic changes (Figure 11). However, untargeted approaches, such as genomics (microarrays or RNAseq), proteomics, or epigenetics are needed to provide a comprehensive understanding of these mechanisms. While such approaches have been applied to other organs like the brain and liver, their use in cardiac research remains limited.

Despite all promising results, limitations exist. Many in vitro studies utilize supraphysiological concentrations of polyphenols, untransformed native molecules instead of circulating metabolites, and unrealistically prolonged exposure times which may not accurately reflect human dietary intake or bioavailability. In vivo studies often focus on short-term interventions, leaving long-term effects and safety profiles underexplored. Moreover, inconsistencies in study designs, including variations in polyphenol types, doses, and models, complicate direct comparisons and meta-analyses.

Future research should prioritize the translation of preclinical findings to clinical settings. Studies employing physiologically relevant doses and exploring chronic interventions are critical. Advanced techniques such as single-cell sequencing and multi-omics approaches can unravel the precise molecular mechanisms and identify patient subgroups most likely to benefit. Addressing the bioavailability challenge through innovative delivery systems, such as nanotechnology or conjugation with bioactive carriers, may enhance therapeutic efficacy. Furthermore, large-scale clinical trials are needed to validate polyphenols’ cardioprotective effects and determine their integration into dietary or pharmaceutical strategies.

## Figures and Tables

**Table 1:** Division of polyphenols and their natural sources.

	Polyphenols	
NON FLAVANOIDS	Class representatives	Food sources
Stilbenes	Resveratol	Grapes, peanuts, berries, wine, nuts, and certain fruits
Lignans	Sekoizolaricirezinol	Red fruits, skin and seeds of blackberries, raspberries, strawberries, black/green tea, oak wood, grape seeds and skins, and pomegranate skins and seeds
Phenolic acids	Caffeine acid, feruine acid, and chlorogenic acid	Red fruit (strawberry, blackberry, raspberry, red apple, and cherry), vegetables (black radish, red onion, and beetroot), tea/coffee, and cereals
Tannins	Hydrolyzable tannins	Red fruits (skin and seeds of blackberries, raspberries, and strawberries), black/green tea, oak wood, grape seeds and skins, pomegranate skins and seeds, and chestnut skins and leaves, as well as dark chocolate, which is enriched with cocoa
FLAVANOIDS		
Flavanones	Narangenin, naringin, hesperetin, hesperedin, and eriodicitol	Grapefruit, orange, mandarin, lime, and lemon
Flavones	Luteolin and apigenin	Spices, yellow or orange fruits and vegetables, parsley, thyme, celery, and hot pepers
Flavonols	Quercetin, myricetin, and kaempferol	Onions, apples, peaches, broccoli, red pepper, grapefruit, and tomatoes, potatoes and nuts
Flavanols	Catechin, and epicatechin epigallocatechin	Berries, dark chocolate, cocoa, apples, strawberries, mango, pear, plum, stone fruits, and black/green tea
Isoflavones	Daidzein, genistein, and daizdin	Soybeans, carrots, green leafy vegetables, broccoli, cabbage, and citrus fruits
Anthocyanidins	Cyanidins, delphinidins, petunidins, and peonidins	Berries, cherries, grapes, pomegranate, red cabbage, and purple sweet potatoes

**Table 2:** Cardioprotective effects and mechanisms of resveratrol in cell models (in vitro).

Cell Model	Concentration	Effects	Mechanisms/ Pathways	References
H9C2 cardiomyoblasts	20 μM	Protection against doxorubicin-induced cytotoxicity, reduced LDH release, oxidative stress markers, and ferroptosis inhibition	MAPK signaling inhibition, and regulation of PTGS2, ACSL4, NCOA4, GPX4, ERK, p38, and JNK	[81]
H9C2 cardiomyoblasts	50 and 100 μM	Increased AMPK and Sirt1 expression, and enhanced cell survival under stress	AMPK activation, and Sirt1-mediated AMPK activation	[82]
Neonatal rat cardiomyocytes	100 μM	Anti-hypertrophic effects, and enhanced PTEN stability	Inhibition of Ang II-induced PTEN degradation, PI3K/AKT/mTOR signaling, and AMPK inactivation	[83]
Cardiomyocytes	1, 3, 5, and 10 µM	Reduced ISO-induced hypertrophy, and decreased hypertrophic markers	Regulation of miR-155/BRCA1 axis	[84]
Neonatal rat cardiomyocytes, HEK293-AT1R	10 µM	Inhibited hypertrophy response, reduced NF-κB signaling, and blocked Ang II/AT1R signaling	Inhibition of ERK 1/2 phosphorylation, and reduced NF-κB p65 phosphorylation	[86]
Neonatal rat ventricular cardiomyocytes	30 μM	Improved viability under hypoxia/reoxygenation	Antioxidant effects	[85]
Neonatal rat cardiac fibroblasts	25 μM	Reduced cardiac fibrosis	Sirt1 activation leading to Smad3 deacetylation	[87,88]

**Table 3:** Cardioprotective effects and mechanisms of quercetin in cell models (in vitro).

Cell Model	Concentration	Effects	Mechanisms/ Pathways	References
H9C2 cardiomyoblasts	0.5, 1, and 2 μM	Reduced hypertrophic markers (ANP, β-MHC), and preserved mitochondrial function	Activation of SIRT3, regulation of SIRT3/PARP-1 pathway, and reduction in oxidative stress	[89]
Neonatal rat cardiomyocytes	20 μM	Decreased hypertrophy, and reduced cell surface area	Inhibition of proteasome activity, activation of GSK-3, and suppression of AKT and AMPK pathways	[90]
Neonatal rat cardiac fibroblasts	100 μM	Inhibited fibrosis markers, reduced fibroblast proliferation and migration, and decreased collagen synthesis	Inhibition of α-SMA, collagen types I and III, fibroblast proliferation, and collagen production	[91]

**Table 4:** Cardioprotective effects and mechanisms of gallic acid in cell models (in vitro).

Cell Model	Concentration	Effects	Mechanisms/ Pathways	References
Neonatal rat cardiomyocytes	10 μM	Reduced hypertrophy, decreased cell size, and downregulated hypertrophic markers (ANP, BNP, and β-MHC)	Promoted autophagy (increased LC3-II/I, ATG5, and ATG6), and inhibition of EGFR, gp130, CaNA, AKT, ERK 1/2, and STAT3 pathways	[92]
Neonatal rat cardiomyocytes	100 mM	Counteracted isoproterenol-induced cell size increase and hypertrophic marker expression	Inhibited β-MHC, ANP, and BNP expression; consistent response across cardiomyocytes and H9C2 cardiomyoblasts	[93]
H9C2 cardiomyoblasts	100 μM	Lowered Nox2 mRNA levels, reduced oxidative stress markers, increased nitric oxide, and decreased MDA	Inhibition of Nox2 via GATA4 modulation, and antioxidative effects (lowered ROS and MDA; and increased NO production)	[94]
Neonatal rat cardiac fibroblasts	Up to 100 μM	Inhibited fibrosis markers (collagen I, fibronectin, CTGF, and α-SMA), and decreased fibroblast activation	Suppression of collagen I expression, fibronectin, CTGF, and α-SMA levels, limiting fibroblast activation and matrix deposition	[95]

**Table 5:** Cardioprotective effects and mechanisms of genistein in cell models (in vitro).

Cell Model	Concentration	Effects	Mechanisms/ Pathways	References
Neonatal rat cardiomyocytes	20 μM	Inhibited phenylephrine-induced hypertrophy, and reduced hypertrophic marker expression	Inhibited JNK1/2 hyper-phosphorylation without altering MAPK and AKT protein levels	[96]
H9C2 cells	10 μM	Reduced isoproterenol-induced hypertrophy, and increased miR-451 expression	miR-451 modulation targeting TIMP2; prevented increase in ANP, BNP, miR-199, and miR-499 associated with hypertrophy	[97]

**Table 6:** Cardioprotective effects and mechanisms of other polyphenols in cell models (in vitro).

Polyphenol	Cell Model	Concentration	Effects	Mechanisms/ Pathways	References
Epigallocatechin-3-gallate and tanshinone IIA sulfonic sodium	H9C2 cardiomyoblasts, and endothelial cells	N/A	Enhanced cell adhesion, and reduced apoptosis and antioxidant protection	Modulated apoptosis, inflammation, and metabolism pathways; and involved NF-κB, TNF, cAMP, and MAPK signaling	[98]
Luteolin	Isolated cardiomyocytes from heart failure rats	16 μM	Improved contractile function, and enhanced peak shortening, contraction, and relaxation rates	Upregulated SERCA2a activity and stability via PI3K/Akt pathway and SERCA2a sumoylation	[99]
Kaempferol	H9C2 cardiomyocytes	25 µM	Reduced cell surface area induced by phenylephrine; and mitigated oxidative stress, inflammation, and apoptosis	Inhibition of ASK1/MAPK pathway; reduction in ROS accumulation; and downregulation of Bcl2 and BA	[100]
Delphinidin	Cardiomyocytes and cardiac fibroblasts	Up to 50 μM	Reduced Ang II-induced hypertrophy, oxidative stress, and fibrotic changes; and decreased cell size and hypertrophic gene expression	Inhibition of NOX activity through AMPK activation; downregulation of NOX subunits (p47phox, Rac1); inhibition of MAPK phosphorylation; and increased AMPK phosphorylation	[101]
Caffeic acid phenethyl ester	H9C2 cardiomyoblasts	20 μM	Reduced phenylephrine-induced hypertrophy; and prevented increase in cell surface area	Inhibition of MEK/ERK pathway; and reduced phosphorylation of MEK1/2 and ERK1/2	[102]

**Table 7:** Cardioprotective effects and mechanisms of resveratrol in animal models (in vivo).

Animal Model	Dosage	Effects	Mechanisms/Pathways	References
Mice (Doxorubicin-induced cardiac dysfunction)	20 mg/kg/day, ip, 2 weeks before doxorubicin	Improved cardiac function (EF, FS), reduced ferroptosis, and preserved myocardial tissue structure	Inhibition of ferroptosis (decreased iron accumulation, restored GSH levels), downregulation of PTGS2, ACSL4, and NCOA4, and upregulation of GPX4	[81]
Mice (Ang II-induced cardiac hypertrophy)	45.51 mg/kg/day, po (oral gavage), 28 days	Reduced cardiac hypertrophy, improved EF and FS, and reduced interstitial fibrosis	Inhibition of NF-κB signaling, and suppression of pro-inflammatory cytokines (TNF-α, IL-6, and IL-1β)	[86]
Mice (Myocardial ischemia–reperfusion injury)	30 mg/kg/day, ip, 7 days	Reduced infarct size, improved cardiac function, reduced oxidative stress, and reduced apoptosis	Activation of AMPK and Sirt1 signaling, enhanced autophagy (LC3B, Beclin-1, and Atg12), decreased ROS, and improved cardiomyocyte survival	[85]
Mice (Heart failure, TAC model)	320 mg/kg/day, po (diet), 3 weeks	Improved survival, reduced LV dilation, enhanced diastolic function, and reduced myocardial fibrosis	Restored AMPK activation, enhanced glucose oxidation, improved mitochondrial function, and increased antioxidant defenses	[103]
Mice (Pressure overload-induced heart failure)	450 mg/kg, po (diet), 2 weeks	Increased exercise capacity, improved glucose utilization, and restored oxidative capacity	Enhanced skeletal muscle insulin signaling (IRS-1, Akt), increased gut microbiome diversity, and metabolic modulation	[104]
Mice (Heart failure with preserved ejection fraction)	10 mg/kg/day, po, 4 weeks	Reduced LV hypertrophy, improved diastolic dysfunction, and reduced fibrosis	Anti-inflammatory effects (IL-1β, IL-6, and TNF-α), anti-oxidative effects (SOD, CAT, and GSH), and inhibition of TGF-β/Smad3 signaling	[88]
Rats (Myocardial infarction-induced heart failure)	2.5 mg/kg/day, po (diet), 16 weeks	Increased LVEF, improved survival, reduced BNP, and enhanced cardiac function	Sirt1 activation, downregulation of proinflammatory and profibrotic markers, and inhibition of CYP1B1	[82]
Rats (Myocardial infarction-induced heart failure)	5.82 mg/kg/day, po (diet), 2 weeks after MI	Improved cardiac function, reduced LV and atrial remodeling, and restored fatty acid oxidation	Energy metabolism restoration, reduction in systolic dysfunction, and inhibition of CYP1B1	[87]
Rats (Isoprenaline-induced myocardial hypertrophy)	20 mg/kg/day, po, 25 day	Reduced cardiac injury markers, improved myocardial architecture, and reduced oxidative stress	Increased GSH, SOD, and CAT, reduced lipid peroxidation, and improved histopathological changes	[105]
Mice (TAC-induced cardiac hypertrophy)	25–50 mg/kg/day, po (oral gavage), 2 weeks	Reduced hypertrophy, restored contractile function, and decreased fibrosis	PTEN stability enhancement, AMPK activation, and downregulation of AKT/mTOR signaling	[83]
Rats (Chronic intermittent hypoxia-induced myocardial hypertrophy)	30 mg/kg/day, po (oral gavage), 5 weeks	Reduced myocardial hypertrophy, improved cardiac function, and reduced apoptosis	Downregulation of PI3K/AKT/mTOR pathway, increased antioxidant enzymes, and enhanced anti-apoptotic signaling	[106]
Mice (TAC-induced cardiac hypertrophy)	150 mg/kg, po (oral gavage), 4 weeks	Alleviated TAC-induced hypertrophy, improved cardiac function, and decreased fibrosis	Modulation of miR-155/BRCA1 pathway, restored LVEF and FS, and reduced hypertrophic markers	[84]
Rats (abdominal aortic banding induced cardiac hypertrophy)	1 mg/kg/day, ip, 14 days	Reduced Ang II levels, and reduced cardiac fibrosis	Downregulation of AT1a mRNA, disruption of RAS signaling, and reduced collagen deposition	[107]

po—per os, ip—intraperitoneally.

**Table 8:** Cardioprotective effects and mechanisms of quercetin in animal models (in vivo).

Animal Model	Dosage	Effects	Mechanisms/Pathways	References
Male spontaneously hypertensive rats	20 mg/kg/day, po (oral gavage), 8 weeks	Reduced cardiac hypertrophy and fibrosis, improved left ventricular function, decreased heart-weight-to-body-weight ratio, and reduced oxidative stress	Activation of SIRT3/PARP-1 pathway, and enhancing mitochondrial protection	[89]
Rats (abdominal aortic constriction—AAC model)	5, 10, and 20 mg/kg/day, po (oral gavage), 8 weeks	Attenuated left ventricular hypertrophy, improved cardiac diastolic function, reduced fibrosis, lower HW/BW ratio, and reduced proteasome activities	Proteasome inhibition and activation of proteasome–glycogen synthesis kinase (GSK)-3α/βUpstream (AKT, and LKB1/AMPKα) and downstream hypertrophic factors (ERK, histone H3, β-catenin, and GATA4)	[90]
Swiss male mice (isoproterenol-induced hypertrophy)	10 mg/kg/day, po, 4 days	Reversed cardiac hypertrophy, restored endogenous antioxidant enzyme activity, reduced oxidative stress, and protected mitochondrial function	Maintained MnSOD activity, and prevented mitochondrial swelling	[109]
C57BL/6 male mice (Ang II-induced hypertrophy)	25 mM/kg, ip, every 2 days for 2 weeks	Reduced cardiac fibrosis and hypertrophy, improved cardiac function, and decreased fibrosis and collagen I and III levels	N/A	[91]

po—per os, ip—intraperitoneally.

**Table 9:** Cardioprotective effects and mechanisms of gallic acid in animal models (in vivo).

Animal Model	Dosage	Effects	Mechanisms/Pathways	References
C57BL/6 male mice (TAC model)	5 or 20 mg/kg/day, po (oral gavage), 8 weeks	Improved cardiac function (EF and FS); reduced cardiomyocyte hypertrophy, fibrosis, and inflammation	Decreased oxidative stress (NOX2, NOX4, and p22phox); reduced inflammatory cytokines (IL-1β, IL-6, andMCP-1); and activated autophagy	[92]
CD-1 male mice (TAC model)	100 mg/kg/day, 2 weeks	Improved cardiac function (reduced LVESD and LVEDD; and normalized FS); reduced HW/BW ratio; and lowered heart failure markers (ANP and BNP)	Downregulated β-MHC and restored α-MHC; reduced fibrosis markers (collagen type I, fibronectin, CTGF, and SMA); and inhibited MMP2 and Smad3	[95]
CD-1 male mice (TAC model)	100 mg/kg/day, ip, 2 weeks	Inhibited TAC-induced increase in left ventricular end-diastolic diameter and left ventricular end-systolic diameter; restored FS; and reduced HW/BW ratio (no effect seen with furosemide)	Inhibition of the expression of epithelial–mesenchymal transition (EMT)-related genes, such as N-cadherin, vimentin, E-cadherin, SNAI1, and TWIST1 in pulmonary tissue	[111]
Male mice (ISP model)	100 mg/kg/day, ip, 4 weeks	Reduced cardiac enlargement, HW/BW, and tibia length ratios; decreased cardiomyocyte area; and improved fractional shortening; reduced fibrosis	Decreased phosphorylation of JNK2 (MAPK pathway); and inhibited Smad3 binding to collagen type I promoter	[93]
Spontaneously hypertensive rats	320 mg/kg/day, po (in drinking water), 16 weeks	Reduced systolic blood pressure; and attenuated LV hypertrophy and myocardial cell size	Suppressed hypertrophic transcription factors GATA4 and GATA6	[94]

po—per os, ip—intraperitoneally

**Table 10:** Cardioprotective effects and mechanisms of genistein in animal models (in vivo).

Animal Model	Dosage	Effects	Mechanisms/Pathways	References
Mouse model subjected to aortic banding (AB) to induce pressure-overload cardiac hypertrophy	40 mg/kg/day, po (oral gavage), 7 weeks	Reduced cardiomyocyte cross-sectional area (CSA), improved heart weight-to-body weight (HW/BW) and lung weight ratios, attenuated hypertrophic markers (ANP, BNP, and β-MHC), preserved α-MHC and SERCA2α expression, decreased cardiac fibrosis, and improved left ventricular function and ejection fraction	Downregulated mRNA of fibrotic markers (CTGF, and collagen types I and III)	[96]
Female ICR mice with isoproterenol-induced cardiac hypertrophy	100 mg/kg, po (diet), 21 days	Reduced cardiac hypertrophy markers, decreased serum malondialdehyde (MDA) levels, increased SOD/MDA ratio, and elevated miR-451 expression	Reduced oxidative stress and enhanced antioxidant capacity	[97]

po—per os.

**Table 11:** Cardioprotective effects and mechanisms of curcumin in animal models (in vivo).

Animal Model	Dosage	Effects	Mechanisms/Pathways	References
New Zealand rabbits (CHF model)	100 mg/kg/day, po (diet), 18 weeks and 3 days	Improved LVEF and LVFS, and reduced left ventricular hypertrophy and fibrosis	Upregulation of DKK-3, inhibition of p38 MAPK and JNK pathways, reduced expression of TNF-α, MMP-2, and MMP-9, and increased SERCA2a expression	[112]
Male Sprague-Dawley rats (ISO-induced hypertrophy)	200 mg/kg/day, po (oral gavage), 4 weeks	Reduced HW/BW ratio, decreased fibrosis, and balanced expression of ANP, MYH6, and MYH7	Downregulation of ANP and MYH7, increased MYH6, inhibition of mTOR-mediated autophagy (decreased LC3, Beclin-1, and increased p-mTOR)	[113]
Male Wistar rats (TAC-induced hypertrophy)	50 mg/kg/day, po (oral gavage), 9 weeks	Improved LVEF and LVFS, reduced HW/BW and LVW/BW ratios, and improved aortic relaxation	Increased expression of NCX and eNOS, and enhanced calcium homeostasis and NO production	[114]
Male septic rats	200 mg/kg/day, ip, 3 days	Improved FS and EF, reduced vacuolar degeneration and fibrosis, and preserved myocardial structure	Decreased cTn I levels, increased SOD, and reduced MDA levels	[115,116]

po—per os, ip—intraperitoneally.

**Table 12:** Standardized mean differences and confidence intervals from two meta-analyses of curcumin’s cardioprotective effects in preclinical models by Zeng et al. 2023 [117] and Li et al. 2023 [118].

Parameter	SMD [117]	95% CI [117]	SMD [118]	95% CI [118]
Myocardial infarction size	−5.65	[−6.94, −4.36]	−17.91	[−22.24, −13.59]
LVEF	2.73	[1.68, 3.79]	10.29	[5.09, 15.48]
LVFS	2.83	[1.83, 3.82]	6.39	[4.27, 8.50]
LV developed pressure	3.59	[2.65, 4.53]	-	-
LV end-diastolic diameter	-	-	−0.43	[−0.74, −0.11]
LV end-systolic diameter	-	-	−1.05	[−1.52, −0.59]
+dP/dt max	3.99	[2.73, 5.25]	-	-
−dP/dt max	3.8	[3.21, 4.40]	-	-
MDA	−7.05	[−11.08, −3.02]	−4.66	[−7.0, −2.31]
SOD	4.92	[3.10, 6.73]	4.47	[1.17, 7.78]
CAT	4.16	[2.64, 5.69]	4.7	[1.48, 7.92]
GSH	-	-	3.66	[0.43, 6.89]
NF-κB	-	-	−5.82	[−9.43, −2.22]
IL-1β	−5.01	[−5.81, −4.22]	-	-
IL-6	−6.67	[−12.36, −0.98]	-	-
TNF-α	−5.62	[−8.50, −2.74]	−4.05	[−6.74, −1.37]
Area of fibrosis	-	-	−2.4	[−4.47, −0.33]
CK	−6.84	[−9.99, −3.68]	−10.93	[−28.62, 6.76]
CK-MB	−3.53	[−5.81, −1.25]	−37.19	[−72.22, −2.16]
LDH	−7.07	[−9.73, −4.40]	−4.29	[−6.01, −2.58]
Apoptotic index	−8	[−14.18, −1.82]	-	-

LVEF—left ventricular ejection fraction; LVFS—left ventricular fractional shortening; LV—left ventricle; +dP/dt max—maximum rate of rise in left ventricular pressure; −dP/dt max—maximum rate of decline in left ventricular pressure; MDA—malondialdehyde; SOD—superoxide dismutase; CAT—catalase; GSH—reduced glutathione; NF-κB—nuclear factor kappa B; IL-1β—interleukin-1 beta; IL-6—interleukin-6; TNF-α—tumor necrosis factor-alpha; CK—creatine kinase; CK-MB—creatine kinase–myocardial band; LDH—lactate dehydrogenase.

**Table 13:** Cardioprotective effects and mechanisms of kaempferol in animal models (in vivo).

Animal Model	Dosage	Effects	Mechanisms/Pathways	References
Male C57BL/6 mice with aortic banding (AB)-induced cardiac hypertrophy	100 mg/kg, po, 6 weeks	Improved systolic and diastolic function; reduced HW/BW and HW/TL ratios, decreased cardiomyocyte size; lowered hypertrophic markers (ANP, BNP, α-MHC, and β-MHC); and reduced interstitial fibrosis and fibrosis markers (fibronectin, CTGF, and collagen I and III)	Modulated TGF-β/Smad and MAPK pathways; inhibited key protein phosphorylation in these pathways; and increased myocardial SOD and glutathione	[100]
Diabetic rats with ISP-induced heart failure	10 mg/kg and 20 mg/kg, po, 42 days	Improved cardiac markers (LDH, troponin-I, and CK-MB); enhanced antioxidant enzymes (SOD, CAT, and glutathione); reduced inflammation (NF-κB, TNF-α, IL-1β, and IL-6) and apoptosis (caspase-3); and decreased lipid peroxidation (MDA)	Activated PI3K/Akt/GSK-3β, and Nrf2; inhibited NF-κB signaling, reducing oxidative stress and inflammation; and increased Bcl-2, supporting cell survival	[119]

po—per os.

**Table 14:** Cardioprotective effects and mechanisms of apigenin in animal models (in vivo).

Animal Model	Dosage	Effects	Mechanisms/Pathways	References
Spontaneously hypertensive rats with infusion into the paraventricular nucleus (PVN)	20 μg/h, infusion directly into the paraventricular nucleus, 4 weeks	Reduced mean arterial pressure and heart rate; decreased cardiac hypertrophy (heart weight, left ventricular weight, and myocardial cell diameter); reduced pro-inflammatory cytokines (TNF-α, IL-6, and MCP-1); enhanced anti-inflammatory cytokine (IL-10); decreased ROS and increased SOD activity in cardiac tissues; and balanced neurotransmitter activity (reduced TH, and increased GAD67)	Inhibition of inflammation and oxidative stress; and reduction in sympathetic tone and cardiac workload through PVN modulation	[120]
Renovascular hypertensive rats induced by two-kidney, one-clip method	50 mg/kg and 100 mg/kg, po (oral gavage), 4 weeks	Lowered blood pressure; reduced heart weight index and cardiomyocyte cross-sectional area; improved myocardial glucolipid metabolism; and reduced serum and myocardial FFA and serum angiotensin II levels	Downregulated HIF-1α, promoting fatty acid oxidation by upregulating PPARα, CPT-1, and PDK-4, while reducing PPARγ and associated genes (GPAT and GLUT-4)	[121]

po—per os.

**Table 15:** Standardized mean differences and confidence intervals from meta-analysis of luteolin cardioprotective effects in preclinical models by Pan et al. 2022 [123].

Parameter	SMD/WMD	95% CI [LAD Ligation]	SMD/WMD	95% CI [Global Ischemia]
Myocardial infarction size	−2.14	[−2.68, −1.59]	−2.87	[−4.72, −1.03]
LVSP	21.62	[18.24, 25.00]	35.40	[29.94, 40.86]
LVEDP	−7.79	[−12.97, −2.61]	−4.73	[−5.90, −3.56]
+dP/dt max	737.48	[521.64, 953.32]	750.47	[623.09, 877.86]
−dP/dt max	605.66	[298.47, 912.84]	790.64	[685.78, 895.49]
MDA	−2.43	[−3.35, −1.51]	-	-
TNF-α	−2.88	[−3.60, −2.16]	-	-
Proportion of apoptotic cells	−11.76	[−12.9, −10.63]	-	-

LVSP—left ventricular systolic pressure; LVEDP—left ventricular end-diastolic pressure; +dP/dt max—maximum rate of rise in left ventricular pressure; −dP/dt max—maximum rate of decline in left ventricular pressure; MDA—malondialdehyde; TNF-α—tumor necrosis factor-alpha.

**Table 16:** Cardioprotective effects and mechanisms of other polyphenols in animal models (in vivo).

Polyphenol	Animal Model	Dosage	Effects	Mechanisms/Pathways	References
Epigallocatechin-3-gallate and Tanshinone IIA sulfonic sodium	New Zealand white rabbits, and Sprague–Dawley rats	Incorporated into glycocalyx-like coatings for implants	Reduced thrombosis, enhanced endothelial cell migration, reduced inflammation in carotid artery implants (rabbits); and decreased fibrosis around subcutaneous implants (rats)	Promotion of endothelial healing, reduction in thrombosis, inflammation regulation, and enhanced biocompatibility of implants	[98]
Luteolin	Sprague–Dawley rats	10 μg/kg/day, ip, 14 days	Improved LV function and contractility, reduced cardiac fibrosis, and decreased oxidative stress and apoptosis	Increased ejection fraction, fractional shortening, reduced collagen deposition, and modulation of oxidative stress, apoptosis (Bax and caspase-3), and structural integrity	[99]
Delphinidin	C57BL/6J mice	15 mg/kg/day, 8 weeks and 6 months in aging mice	Reduced cardiac hypertrophy, restored LV function, decreased ROS and NOX activity, and reduced fibrosis	Modulation of hypertrophic gene expression (Anp, Bnp, and β-MHC), reduced ROS and MAPK inhibition (Erk1/2, Jnk1/2, and p38), and increased AMPK phosphorylation and anti-fibrotic effects	[101]
Caffeic Acid Phenethyl Ester	Mice, and murine model of pressure overload (aortic banding)	100 mg/kg/day, po (oral gavage), 6 weeks	Reduced cardiac hypertrophy, improved cardiac function, and attenuated fibrosis	Inhibition of MEK/ERK and TGF-β/Smad signaling, reduced collagen deposition, and restored cardiac function	[102]
Hesperidin	Rat model with ISO-induced myocardial injury	200 mg/kg/day po, 28 days	Preserved heart structure, reduced hypertrophy, normalized hemodynamics, and reduced oxidative stress and fibrosis	Antioxidant properties, reduced MDA, CK-MB, and LDH, increased GSH, SOD, CAT, reduced caspase-3, and increased Bcl-2, and modulation of apoptotic pathways and inflammatory responses	[124]
Pterostilbene	Rat model of pulmonary arterial hypertension (MCT)	25, 50, or 100 mg/kg, po (oral gavage), 14 days	Dose-dependent cardioprotection, reduced oxidative stress, and improved RV function	Antioxidant mechanisms, upregulation of SERCA, restored GSH/GSSG ratio, reduced phospholamban, enhanced antioxidant enzyme activities, and reduced myocardial oxidative damage	[125]
Punicalagin	LDLR −/− mice with high-fat diet (HFD)	250 mg/kg or 500 mg/kg, po (oral gavage), 12 weeks	Reduced atherosclerotic plaques, improved endothelial function, and decreased inflammation, and fibrosis	Reduction in lipid metabolism disorders, anti-inflammatory effects (macrophage infiltration, and cytokine suppression), enhancement of nitric oxide bioavailability, and inhibition of Smad1/5 signaling	[126]

po—per os, ip—intraperitoneally.
